# Inhibition of NFAT5‐Dependent Astrocyte Swelling Alleviates Neuropathic Pain

**DOI:** 10.1002/advs.202302916

**Published:** 2024-01-09

**Authors:** Liqiong He, Shengyun Ma, Zijin Ding, Zhifeng Huang, Yu Zhang, Caiyun Xi, Kailu Zou, Qingwei Deng, Wendy Jia Men Huang, Qulian Guo, Changsheng Huang

**Affiliations:** ^1^ Department of Anesthesiology Xiangya Hospital Central South University Changsha 410008 China; ^2^ Department of Cellular and Molecular Medicine University of California San Diego San Diego CA 92093 USA; ^3^ National Clinical Research Center for Geriatric Disorders Xiangya Hospital Central South University Changsha 410008 China

**Keywords:** AQP4, astrocyte swelling, AURKB, neuropathic pain, NFAT5

## Abstract

Astrocyte swelling is implicated in various neurological disorders. However, whether astrocyte swelling contributes to neuropathic pain remains elusive. This study elucidates the pivotal role of the nuclear factor of activated T‐cells 5 (NFAT5) emerges as a master regulator of astrocyte swelling in the spinal dorsal horn (SDH) during neuropathic pain. Despite the ubiquitous expression of NFAT5 protein in SDH cell types, it selectively induces swelling specifically in astrocytes, not in microglia. Mechanistically, NFAT5 directly controls the expression of the water channel aquaporin‐4 (AQP4), a key regulator exclusive to astrocytes. Additionally, aurora kinase B (AURKB) orchestrates NFAT5 phosphorylation, enhancing its protein stability and nuclear translocation, thereby regulating AQP4 expression. The findings establish NFAT5 as a crucial regulator for neuropathic pain through the modulation of astrocyte swelling. The AURKB‐NFAT5‐AQP4 pathway in astrocytes emerges as a potential therapeutic target to combat neuropathic pain.

## Graphical abstract

Peripheral nerve injury induces morphological changes in spinal dorsal horn astrocytes, characterized by significant cell swelling, a prominent pathological feature in the context of neuropathic pain. The NFAT5 signaling pathway plays a crucial role in astrocyte swelling after peripheral nerve injury. AURKB mediates NFAT5 phosphorylation, promotes NFAT5 translocation into the nucleus and protects it from degradation. The nuclear NFAT5 further stimulates the transcription and expression of AQP4, which subsequently leads to astrocyte swelling and contributes to the development of neuropathic pain.

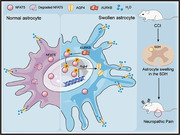

## 1 Introduction

Neuropathic pain affects as many as 7–10% of the general population, but the treatment options remain limited even though many approaches have been attempted.^[^ ^1^ ^]^ Neuropathic pain is caused by the damage or lesion of the somatosensory system, including peripheral nerves, spinal cord, and brain. The induction and maintenance of neuropathic pain states involve ectopic activity in the dorsal root ganglia (DRG) and the spinal dorsal horn (SDH),^[^ ^2^ ^]^ then leading to peripheral and central sensitization including activation of spinal glia and neurons to release excitatory and neuroinflammatory factors, and thus contribute to the establishment of long‐term pain states.^[^ ^3^, ^4^ ^]^ Astrocytes constitute ≈30% of the cells in the mammalian central nervous system (CNS), and they are integral to brain and spinal cord physiology and perform many functions important for normal neuronal development, synapse formation, and proper propagation of action potentials.^[^ ^5^, ^6^, ^7^ ^]^ Under physiological conditions, astrocytes provide structural and metabolic support to neurons and help to maintain physiological levels of various extracellular ions, glutamate, and water.^[^ ^8^ ^]^ However, peripheral nerve injury or chemotherapy‐induced neuropathy induces significant injury induces reactive astrocytes or astrogliosis, causing deleterious morphological and functional changes.^[^ ^9^, ^10^ ^]^ These changes, including the release of inflammatory mediators, drive neuropathic pain by inducing synaptic and neuronal plasticity.^[^ ^6^, ^7^, ^10^, ^11^, ^12^ ^]^

Reactive astrocytes display significant molecular, proliferative, morphological, and functional changes during neuropathic pain.^[^ ^13^ ^]^ They rapidly proliferate at lesion sites, expressing increased glial fibrillary acidic protein (GFAP), a major protein component of astrocyte intermediate filaments.^[^ ^13^, ^14^ ^]^ Morphological alterations, such as hypertrophy and increased processes, play a crucial role in chronic pain and CNS disease.^[^ ^12^, ^15^, ^16^, ^17^ ^]^ Notably, the excessive influx of water that leads to the swelling of astrocytes contributes significantly to the morphological changes observed in reactive astrocytes during CNS injuries such as cerebral edema, stroke, and traumatic brain injury.^[^ ^18^, ^19^, ^20^ ^]^ However, it remains largely unknown whether peripheral nerve injury induces astrocyte swelling, contributing to morphological changes. Investigating the mechanisms behind astrocyte morphological changes post peripheral nerve injury could open novel avenues for neuropathic pain treatment.

The nuclear factor of activated T‐cells (NFAT) is known for its role in the immune response. This family has 5 members, NFAT1‐5, that all share a conserved DNA‐bind domain. NFAT1‐4 has been reported to be correlated or contributed to neuropathic pain,^[^ ^21^, ^22^, ^23^, ^24^ ^]^ especially NFAT1 can promote neuropathic pain by activating microglial proliferation.^[^ ^24^ ^]^ NFAT5, less studied in this context, plays a crucial role in cell tonicity and volume regulation, thus is also named as tonicity‐responsive enhancer binding protein (TonEBP) .^[^ ^25^ ^]^ When exposed to hypertonic stress, the NFAT5‐regulated expression of specific transporters leads to uptake of osmolytes such as betaine, taurine, and myo‐inositol, which increases the cell volume from the hyperosmolar stress‐triggered cell shrinkage.^[^ ^25^ ^]^ Moreover, NFAT5 is necessary for transcriptional regulation of several aquaporins which are essential for cell volume regulation.^[^ ^18^, ^26^, ^27^ ^]^ Distinct from its role in osmotic adaptation, NFAT5 is upregulated in cytotoxic edemainduced hippocampal hypertrophic astrocytes and regulates AQP4 expression in an ammonia‐induced swelling model of astrocytes in vitro.^[^ ^26^ ^]^ NFAT5 haplodeficiency in vivo and knockdown in vitro attenuate astrocyte‐associated neuroinflammation.^[^ ^28^, ^29^ ^]^ These literatures suggest a role of NFAT5 in astrocyte‐associated neuroinflammation.

Based on these, we hypothesized NFAT5's contribution to reactive astrocyte morphological changes and neuropathic pain. Using the chronic constriction injury (CCI) model, we demonstrated that genetic or pharmacological intervention of NFAT5 specifically attenuated reactive astrocyte swelling in the SDH and alleviated rat's neuropathic pain behaviors. Specifically, NFAT5 positively regulates the transcription of *Aqp4*, the dominant water channel protein specifically in astrocytes, and targeting NFAT5‐AQP4 signaling alleviated astrocyte swelling and the development of neuropathic pain. Furthermore, we found that the serine/threonine protein kinase aurora kinase B (AURKB) phosphorylates NFAT5, promoting its nuclear localization and protein stability postCCI. The AURKB‐NFAT5‐AQP4 pathway in astrocytes provides potential targets for drug development to control neuropathic pain.

## 2 Results

### 2.1 Genetic Knockdown or Pharmacological Inhibition of NFAT5 Alleviate CCI induced Neuropathic Pain

**Figure 1:**
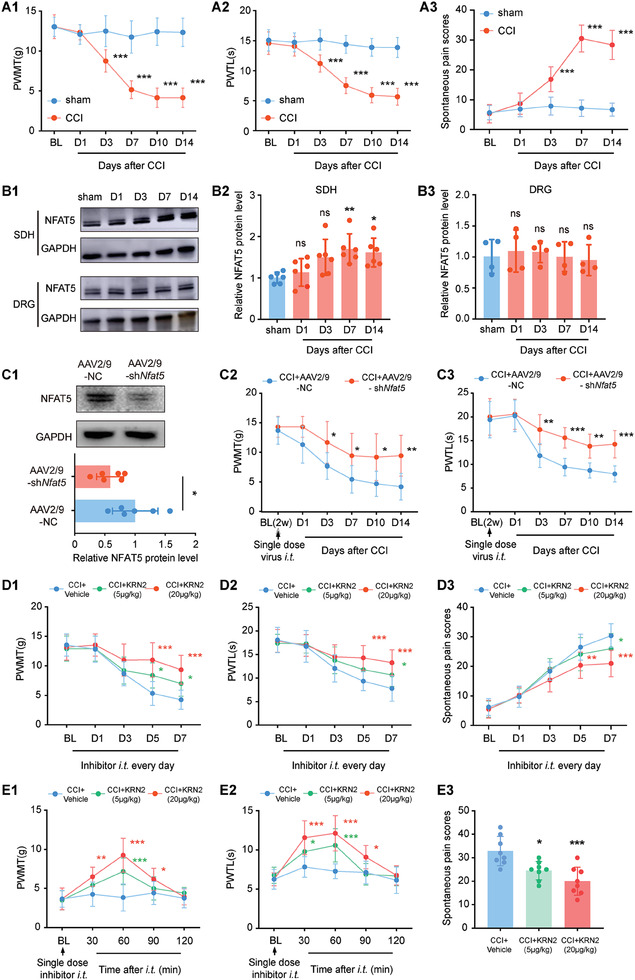
Targeting NFAT5 alleviated CCI‐induced neuropathic pain. A1‐A3) Behavioral tests of paw withdrawal mechanical threshold (PWMT), paw withdrawal thermal latency (PWTL), and spontaneous pain scores after CCI surgery. ***p < 0.001, *n* = 8, two‐way ANOVA with repeated measures followed by Tukey's multiple comparison test. B1‐B3) NFAT5 protein level in the injured ipsilateral spinal dorsal horn (SDH) and ipsilateral L4‐L5 DRG after sham or CCI surgery. *p < 0.05, **p < 0.01, *n* = 6, one‐way ANOVA followed by Tukey's multiple comparison test. C1) Validation of knockdown efficiency after intrathecal (*i.t*.) injection of AAV2/9‐sh*Nfat5* in rats SDH. *p < 0.05, *n* = 6, two‐tailed unpaired student's *t*‐test. C2‐C3) Rats were *i.t*. injection of AAV2/9‐sh*Nfat5* for 2 weeks followed by CCI modeling and then performed PWMT and PWTL pain behavioral tests. CCI + AAV2/9‐sh*Nfat5* versus CCI + AAV2/9‐NC, *p < 0.05, **p < 0.01, ***p < 0.001, *n* = 8, two‐way ANOVA with repeated measures followed by Tukey's multiple comparison test. D1‐D3) Behavioral tests of PWMT, PWTL and spontaneous pain scores after daily *i.t*. administration of the NFAT5 inhibitor KRN2 for 7 days after CCI modeling. CCI + KRN2 (5 µg kg^−1^) versus CCI + Vehicle, *p < 0.05, *n* = 8, CCI + KRN2 (20 µg kg^−1^) versus CCI + Vehicle, **p < 0.01, ***p < 0.001, *n* = 8, two‐way ANOVA with repeated measures followed by Tukey's multiple comparison test. E1‐E2) Behavioral tests of PWMT and PWTL after single *i.t*. administration of the NFAT5 inhibitor KRN2 on day 7 CCI‐treated rats. CCI + KRN2 (5 µg kg^−1^) versus CCI + Vehicle, *p < 0.05, ***p < 0.001, *n* = 8, CCI + KRN2 (20 µg kg^−1^) versus CCI + Vehicle, *p < 0.05, **p < 0.01, *** *p* < 0.001, *n* = 8, two‐way ANOVA with repeated measures followed by Tukey's multiple comparison test. E3) Behavioral tests of spontaneous pain scores after single *i.t*. administration of the NFAT5 inhibitor KRN2 on day 7 CCI‐treated rats. CCI + KRN2 (5 µg kg^−1^) versus CCI + Vehicle, *p < 0.05, *n* = 8, CCI + KRN2 (20 µg kg^−1^) versus CCI + Vehicle, ***p < 0.001, *n* = 8, one‐way ANOVA followed by Tukey's multiple comparison test.

To investigate the involvement of NFAT5 in neuropathic pain, we assessed its protein expression in the CCI induced neuropathic pain model. After CCI surgery, we evaluate two main symptoms of neuropathic pain—evoked pain and spontaneous pain—using the paw withdrawal mechanical threshold (PWMT), the paw withdrawal thermal latency (PWTL), and the spontaneous pain scores, confirming that the induction of neuropathic pain in rats (**Figure** **1** A1–A3). Subsequently, we examined NFAT5 protein levels in the injured ipsilateral SDH and ipsilateral L4‐L5 DRG. As depicted in Figure 1B1–B3, NFAT5 protein levels showed a continuous increase in the ipsilateral SDH of CCI rats, peaking at day 7 post‐surgery and maintaining a high level until day 14. Interestingly, this trend was not observed in the ipsilateral L4‐L5 DRG. We further characterized NFAT5‐expressing cells by co‐staining the NFAT5 antibody with the neuronal marker NeuN, the astrocyte marker GFAP, or the microglia marker IBA1 in the SDH. NFAT5 was found to be widely distributed in all the cell types both in the sham and CCI group, consistent with previous studies^[^ ^30^, ^31^, ^32^ ^]^ (Table S2, Supporting Information). Notably, the distribution of NFAT5 in microglia and astrocytes in the SDH changed after CCI: increasing from 15% to 21% in microglia, and from 19% to 28% in astrocytes (Figure S1A1‐A3, Supporting Information). Collectively, these data suggested that NFAT5 protein is upregulated in astrocytes and microglia during neuropathic pain.

To understand the role of NFAT5 in neuropathic pain, the established AAV2/9 of sh*Nfat5* (AAV2/9‐sh*Nfat5*) was delivered via an intrathecal catheter to the SDH two weeks prior to CCI surgery (Figure 1C1; Figure S1B1, Supporting Information). Immunofluorescence staining confirmed the non‐specific transfection of AAV2/9‐sh*Nfat5* into neurons, microglia, and astrocytes of the SDH (Figure S1B2, Supporting Information). Compared with the vehicle group (AAV2/9‐NC), rats treated with AAV2/9‐sh*Nfat5* showed relieved mechanical allodynia and heat hyperalgesia from day 3 until day 14 after CCI (Figure 1C2–C3), suggesting NFAT5 is necessary for CCI‐induced neuropathic pain. Similarly, sustained inhibition of NFAT5 with its inhibitor KRN2 ameliorated both evoked and spontaneous pain in CCI rats in a dose‐dependent effect (Figure 1D1–D3). Remarkably, even a single dose administration of KRN2 on day 7 after CCI surgery rapidly relieved neuropathic pain, with effects observed as early as 30 minutes post‐administration and lasting for ≈1 h (Figure 1E1–E3). Altogether, these data demonstrated that NFAT5 is a crucial factor in CCI‐induced neuropathic pain, and targeting NFAT5 provides relief from this condition.

### 2.2 Targeting NFAT5 Attenuates Swelling of Astrocytes During Neuropathic Pain

**Figure 2:**
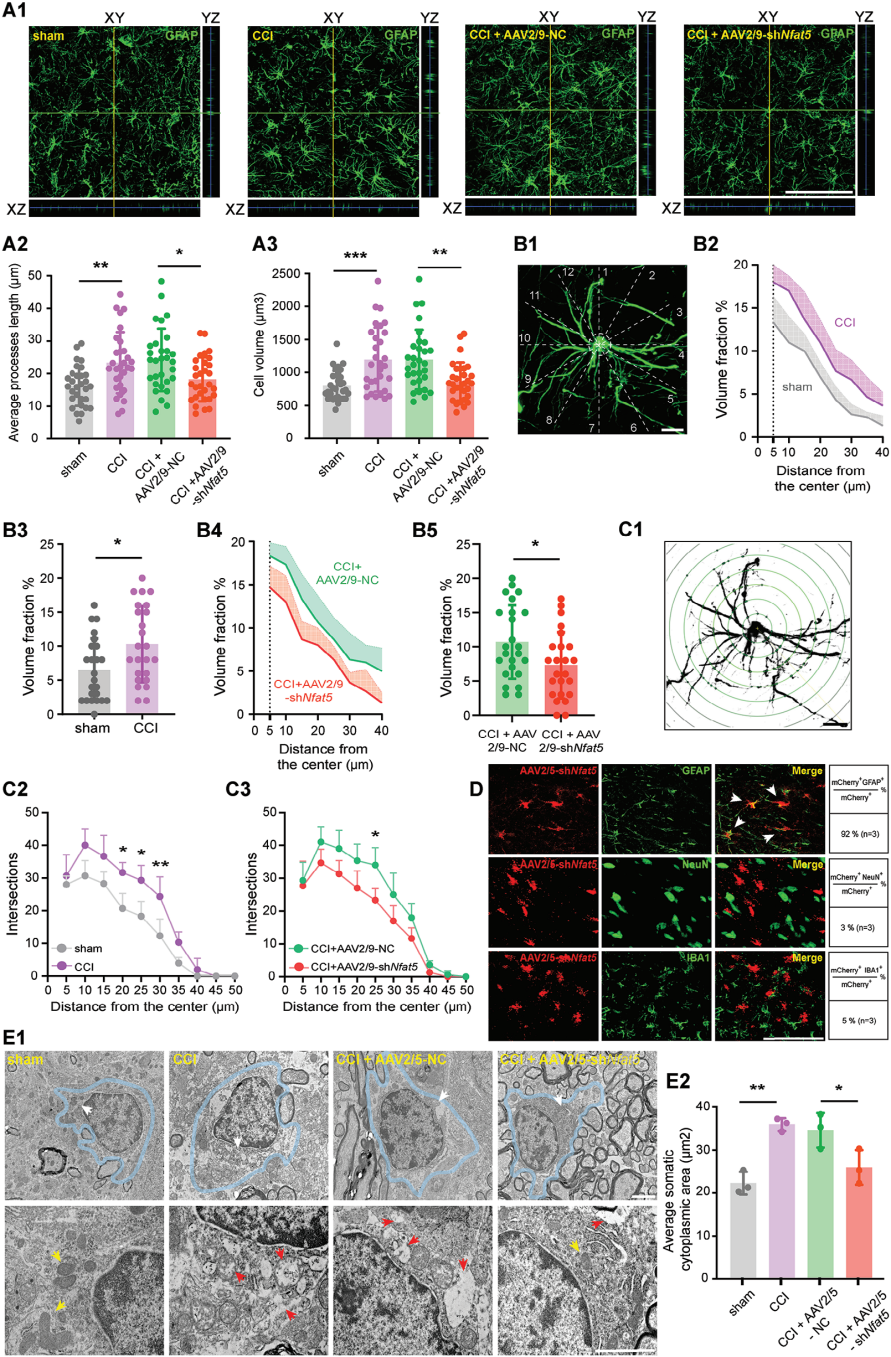
Targeting NFAT5 attenuates astrocyte swelling during neuropathic pain. A1) Representative 3D orthogonal confocal images of GFAP‐stained astrocytes in indicated group of rats SDH. Scale bar = 100 µm. A2‐A3) Morphological changes (average processes length and cell volume) of astrocytes in the SDH. CCI versus sham, **p < 0.01, ***p < 0.001, CCI + AAV2/9‐NC versus CCI + AAV2/9‐sh*Nfat5*, *p < 0.05, **p < 0.01, *n* = 30 (3 rats per group, and 10 typical cells per rat were analyzed), one‐way ANOVA followed by Tukey's multiple comparison test. B1) Schematic diagram of astrocytes processes volume fraction estimation, scale bar = 10 µm. B2‐B5) Estimation of astrocytes processes volume fraction. CCI versus sham, *p < 0.05, CCI + AAV2/9‐NC versus CCI + AAV2/9‐sh*Nfat5*, *p < 0.05, *n* = 24 (3 rats per group, and 8 volume fractions of each rat ranged from 5 to 40 µm were analyzed), two‐tailed unpaired student's *t*‐test. C1) Schematic diagram of astrocytes Sholl analysis, scale bar = 10 µm. C2‐C3) Sholl analysis of astrocytes. CCI versus sham, *p < 0.05, **p < 0.01, *n* = 3, CCI + AAV2/9‐NC versus CCI + AAV2/9‐sh*Nfat5*, *p < 0.05, *n* = 3, two‐way ANOVA followed by Tukey's multiple comparison test. D) Transfection efficiency of intra‐spinal injection of AAV2/5‐sh*Nfat5* in the indicated cell types. Quantification of transfection efficiency was shown on the right (pooled from 3 rats/group). Scale bar = 100 µm. E1‐E2) Representative transmission electron microscopy images and average area of the somatic cytoplasm statistics of astrocytes in indicated group of rats SDH after intra‐spinal injection of AAV2/5‐sh*Nfat5*. The area within the blue curve indicates astrocytes, the white arrow in the upper panel image indicates the area that is magnified in the lower panel of the image, the yellow arrow indicates cytoplasm or mitochondria without swelling, and the red arrow indicates cytoplasm or mitochondria with obvious swelling. Scale bar = 2 µm. CCI versus sham, **p < 0.01, CCI + AAV2/5‐NC versus CCI + AAV2/5‐sh*Nfat5*, *p < 0.05, *n* = 3, one‐way ANOVA followed by Tukey's multiple comparison test.

In the context of neuropathic pain, astrocytes and microglia undergo activation accompanied by morphological changes in the SDH.^[^ ^12^, ^33^ ^]^ Consistently, astrocytes exhibited increased processes length and cell volume on day 7 post‐CCI, while microglia displayed decreased processes length and increased cell volume (**Figure** **2**A1–A3; Figure S2A1–A3, Supporting Information). Interestingly, knockdown of NFAT5 reversed the morphological changes caused by CCI surgery in the astrocytes but not in microglia (Figure 2A1–A3; Figure S2A1–A3, Supporting Information). Detailed morphological changes of astrocytes were further observed under high magnification. To quantify the changes in astrocyte processes volume after CCI, their volume fractions were analyzed as previously described.^[^ ^34^, ^35^ ^]^ CCI significantly increased the volume fraction of astrocyte processes, a change that was reversed by NFAT5 knockdown (Figure 2B1–B5). Sholl analysis results also suggested that the number of astrocyte processes at different distances from the soma significantly increased after CCI, a change that was reversed by NFAT5 knockdown (Figure 2C1–C3). In addition, the role of NFAT5 in astrocyte morphological changes was tested in the rat astrocyte cell line CTX‐TNA2 using Calcein AM, an indicator of the intracellular space.^[^ ^36^, ^37^ ^]^ NFAT5 inhibition reversed the LPS‐induced increase in fluorescence intensity, indicating a reduction in cell swelling (Figure S3A1–A2, Supporting Information). These findings collectively suggest that NFAT5 specifically regulates astrocyte morphology.

To validate the NFAT5‐dependent astrocyte‐specific function, NFAT5 knockdown AAV viruses carrying specific astrocyte promoters (AAV2/5‐sh*Nfat5*) were designed according to previous studies.^[^ ^38^ ^]^ The microscopic intraspinal injection was used to avoid potential DRG interference from intrathecal injection (Figure S3B1–B2, Supporting Information). Immunofluorescence staining and western blot experiments confirmed the specific transfection of AAV2/5‐sh*Nfat5* to astrocytes, leading to the successful knockdown of NFAT5 (Figure 2D; Figure S3B3, Supporting Information). In line with our previous results, astrocyte‐specific downregulation of NFAT5 relieved mechanical allodynia and heat hyperalgesia after CCI surgery (Figure S3C1–C2, Supporting Information), as well as reduced processes length and cell volume of the astrocytes (Figure S3D1–D3, Supporting Information). To further observe CCI‐induced morphological responses in astrocytes, microscopic morphological structural changes of astrocytes in the SDH were examined using transmission electron microscopy (TEM). Under the TEM, astrocytes showed an oval nuclear outline with a relatively uniform and low density of nucleoplasm and cytoplasm, and a thin and dense heterochromatin rim under the nuclear membrane. Organelles such as endoplasmic reticulum, free glycogen granules, and mitochondria were sparsely distributed in the cytoplasmic stroma, and their cytoplasmic and mitochondrial membranes had a lower electron density than neurons and microglia.^[^ ^39^ ^]^ TEM imaging revealed significant morphological changes in SDH astrocytes, including swelling of the cell body on day 7 after CCI (the area within the blue curve in Figure 2E1), and the mitochondria in the cells also became swollen after CCI, showing disorganized or missing cristae and vacuolation. These changes were reversed by specific knockdown of NFAT5 in SDH astrocytes (Figure 2E1–E2). In contrast, SDH microglia did not show significant swelling in sham or CCI groups (Figure S2B, Supporting Information).

To investigate potential gender differences in the regulation of NFAT5 in neuropathic pain and morphological changes in the SDH, validation experiments were also conducted in female rats. Similar to male rats, NFAT5 protein expression was consistently upregulated in the SDH after CCI modeling in female rats. Continuous inhibition of NFAT5 with inhibitor KRN2 also relieved evoked and spontaneous pain in female CCI rats (Figure S4A1–C3, Supporting Information). Importantly, inhibition of NFAT5 in female rats also reversed astrocyte swelling induced by CCI surgery (Figure S4D1–D3, Supporting Information). Taken together, these results suggested that NFAT5 regulates the swelling of SDH astrocytes during neuropathic pain in a gender‐independent manner.

### 2.3 NFAT5 Regulates the Expression of the Astrocytic Marker AQP4

**Figure 3:**
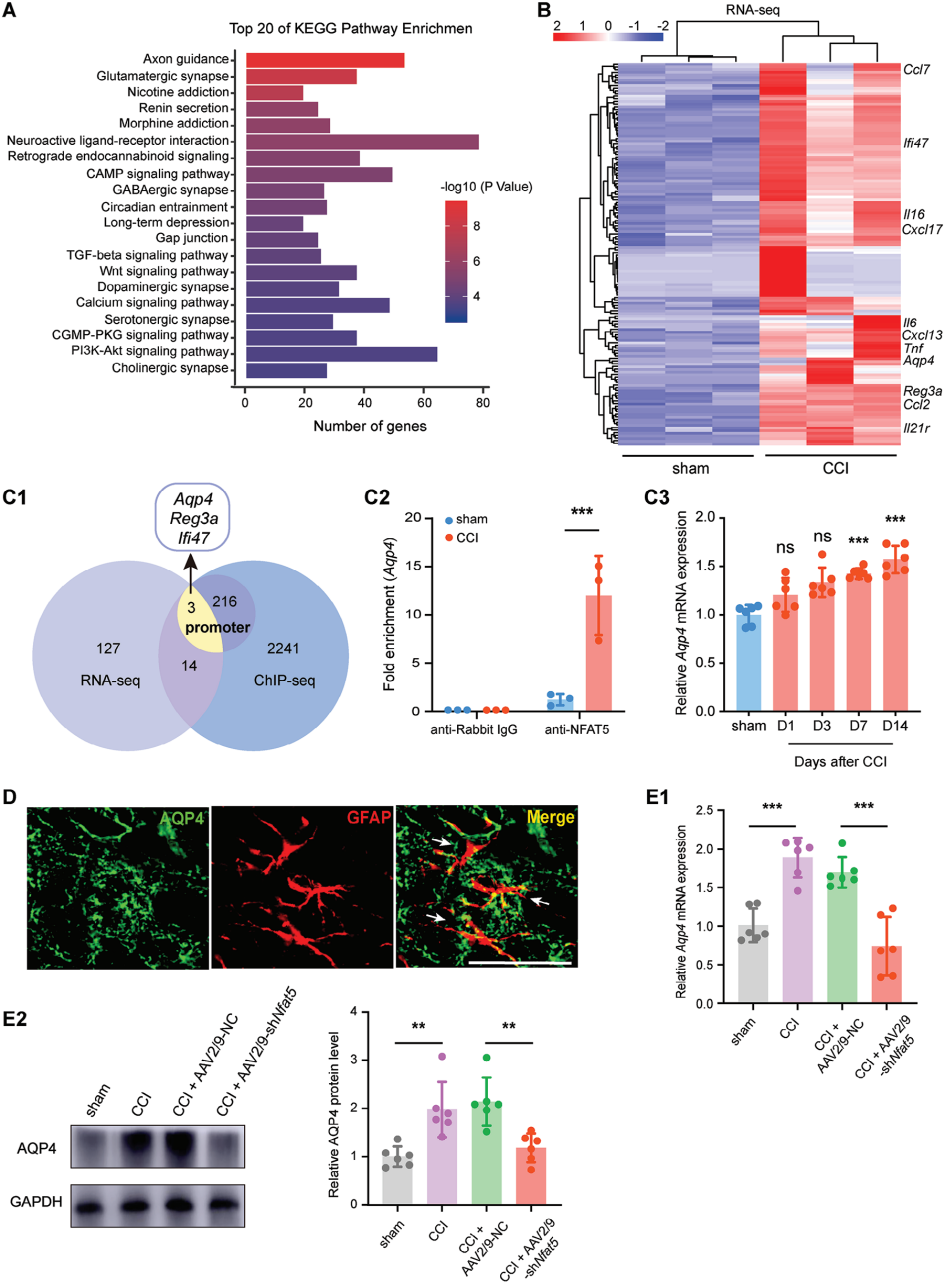
NFAT5 regulates AQP4 expression. A) Kyoto Encyclopedia of Genes and Genomes (KEGG) enrichment analysis of ChIP‐seq of NFAT5 targets. B) Heat map showed 144 significantly up‐regulated genes in the SDH of CCI group rats compared with the sham group by RNA‐seq analysis. C1) The intersection of NFAT5 ChIP‐seq data with RNA‐seq data from sham and CCI groups. C2) ChIP‐qPCR analysis of endogenous NFAT5 binding at *Aqp4* promoters in the SDH after CCI. ***p < 0.001, *n* = 3, two‐tailed unpaired Student's *t*‐test. C3) Expression of *Aqp4* mRNA in the injured ipsilateral SDH after sham or CCI surgery. Compared to the sham‐operated rats, ***p < 0.001, *n* = 6, one‐way ANOVA followed by Tukey's multiple comparison test. D) Immunofluorescence double‐labeling of AQP4 (green) and astrocyte marker GFAP (red) in the SDH on day 7 after CCI modeling. Scale bar = 100 µm. E1) *Aqp4* mRNA expression after *i.t*. administration of AAV2/9‐sh*Nfat5* in the SDH of CCI rats. CCI versus sham, ***p < 0.001, CCI + AAV2/9‐sh*Nfat5* versus CCI + AAV2/9‐NC, ***p < 0.001, *n* = 6, one‐way ANOVA followed by Tukey's multiple comparison test. E2) AQP4 protein expression after *i.t*. administration of AAV2/9‐sh*Nfat5* in the SDH of CCI rats. CCI versus sham, **p < 0.01, CCI + AAV2/9‐sh*Nfat5* versus CCI + AAV2/9‐NC, **p < 0.01, *n* = 6, one‐way ANOVA followed by Tukey's multiple comparison test.

To identify the direct targets of NFAT5, we performed chromatin immunoprecipitation sequencing (ChIP‐seq) of NFAT5 in the SDH on the sham group and CCI group rats (Table S3, Supporting Information). Consistent with previous publications,^[^ ^40^ ^]^ NFAT5 exhibited preferential enrichment in intergenic and intronic regions, and this binding preference was unaffected by CCI surgery (Figure S5A1–A2, Supporting Information). Differential analysis revealed 4722 NFAT5 binding peaks near 2474 gene loci, and Kyoto Encyclopedia of Genes and Genomes (KEGG) enrichment analysis indicated a preference for astrocytic responsive pathways, including glutamatergic synapse,^[^ ^41^ ^]^ Gap junction^[^ ^42^ ^]^ and Wnt signaling pathway^[^ ^43^ ^]^ (**Figure** **3**A). To investigate CCI‐induced transcriptomic alterations, RNA sequencing (RNA‐seq) was performed on SDH tissues from sham and CCI group rats (Table S4, Supporting Information), revealing 144 significantly upregulated genes on day 7 post‐CCI surgery (cutoff: log_2_FC ≥ 1.5, P‐value ≤ 0.05) (Figure 3B; Figure S5B1–B2, Supporting Information). Validation confirmed the upregulation of inflammatory factors (*Tnf*, *Il6*, *Il1b*) and chemokines (*Ccl2* and *Cxcl1*) (Figure S5C1–C5, Supporting Information), both are closely associated with astrocyte regulation of chronic pain.^[^ ^10^, ^44^ ^]^ Notably, inhibition of NFAT5 reversed these trends, suggesting a potential association between NFAT5 and astrocyte‐related changes in CCI‐induced neuropathic pain (Figure S5C1–C5, Supporting Information).

Integration of NFAT5 binding targets with differentially expressed genes identified 17 transcripts, notably, this list contains the astrocyte‐specific marker *Aqp4* (Figure 3C1; Table S5, Supporting Information), the dominant water channel protein in the CNS, which is involved in regulating astrocytic functions and participating in many diseases of the central nervous system,^[^ ^18^, ^45^ ^]^ and highly associated with astrocyte swelling.^[^ ^18^ ^]^ NFAT5 is directly bound to the *Aqp4* promoter, and this binding was enhanced after CCI surgery (Figure 3C1–C2; Figure S5D, Supporting Information). Additionally, *Aqp4* transcription increased with neuropathic pain development (Figure 3C3). Immunofluorescence staining confirmed astrocytic‐specific expression of AQP4 in the SDH, with increased AQP4 protein levels after CCI surgery, a change reversed by NFAT5 knockdown (Figure 3D,E; Figure S5E,F, Supporting Information). Thus, these data suggested that NFAT5 directly controls AQP4 expression in astrocytes during neuropathic pain.

### 2.4 Inhibition of AQP4 Ameliorates CCI‐Induced Neuropathic Pain and Astrocyte Swelling

**Figure 4:**
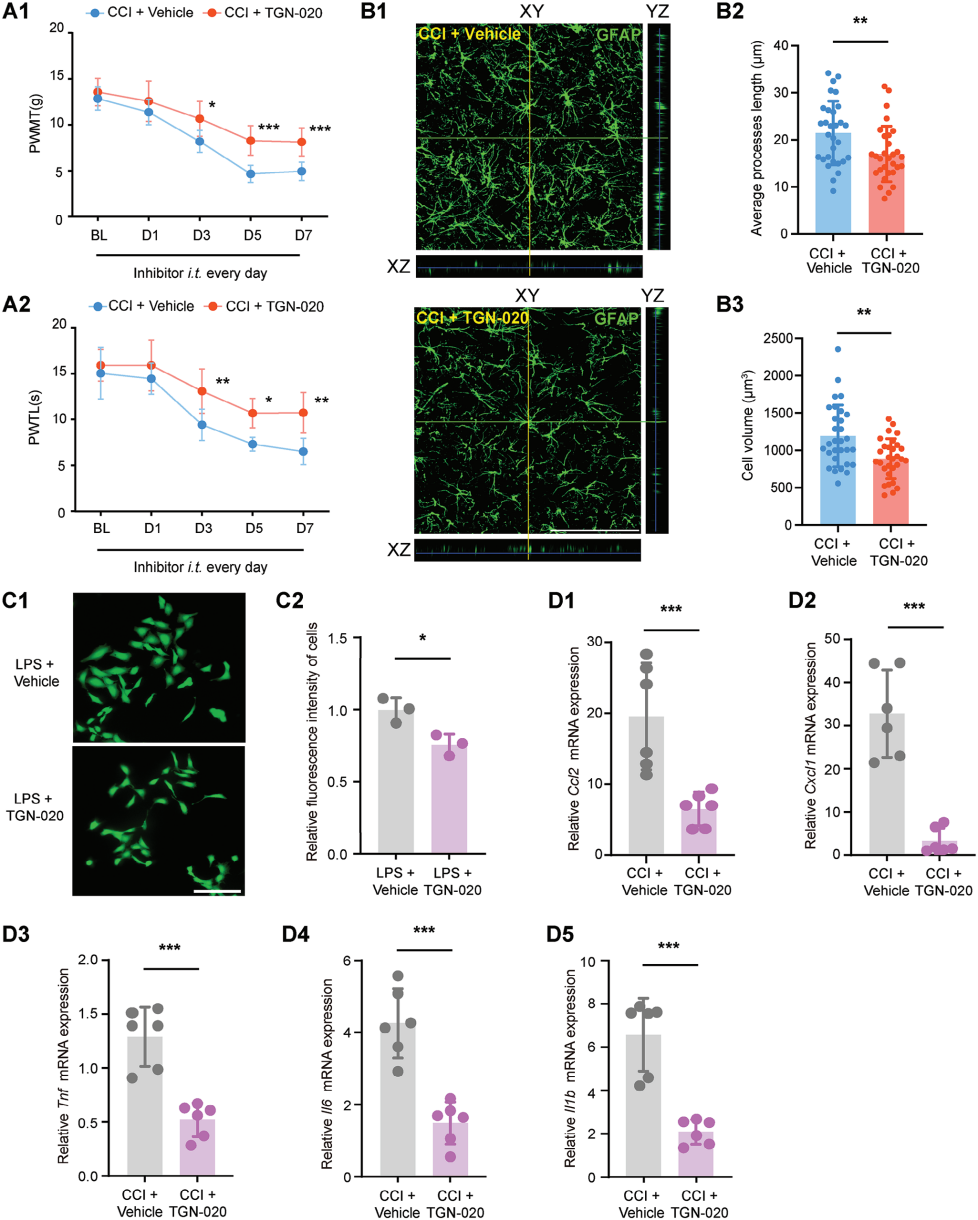
AQP4 inhibition ameliorates CCI‐induced neuropathic pain and astrocyte swelling. A1‐A2) Behavioral tests of PWMT and PWTL after daily *i.t*. administration of the AQP4 inhibitor TGN‐020 over 7 days after CCI modeling. *p < 0.05, **p < 0.01, ***p < 0.001*, n* = 8, two‐way ANOVA with repeated measures followed by Tukey's multiple comparison test. B1) Representative 3D orthogonal confocal images of GFAP‐stained astrocytes in each treatment group of rats SDH after *i.t*. administration of AQP4 inhibitor. Scale bar = 100 µm. B2‐B3) Morphological changes (average processes length and cell volume) of GFAP‐stained astrocytes in each treatment group of rats SDH after *i.t*. administration of AQP4 inhibitor. **p < 0.01, *n* = 30 (3 rats per group, and 10 typical cells per rat were analyzed), two‐tailed unpaired student's *t*‐test. C1‐C2) Representative fluorescence images of CTX‐TNA2 cells Calcein AM permeation assay in vitro and relative mean fluorescence intensity statistics of cells. *p < 0.05, *n* = 3, two‐tailed unpaired student's *t*‐test. D1‐D5) Indicated mRNA expression in the SDH after daily intrathecal administration of AQP4 inhibitor in CCI rats. ***p < 0.001, *n* = 6, two‐tailed unpaired student's *t*‐test.

To explore the function of AQP4 in CCI‐induced neuropathic pain, AQP4‐specific inhibitor TGN‐020 was continuously administered intrathecally for 7 days after CCI surgery. TGN‐020 recipient rats experienced increased mechanical allodynia and heat hyperalgesia, along with attenuated morphological changes in astrocytes within the SDH (**Figure** **4**A1–B3). In vitro administration of TGN‐020 on CTX‐TNA2 astrocytes also reduced Calcein AM fluorescence intensity, indicating a role for AQP4 in astrocyte swelling (Figure 4C1–C2). These data suggested that AQP4 is required for neuropathic pain and morphological swelling in astrocytes.

Recent studies have shown that AQP4 regulates astrocyte‐mediated neuroinflammation.^[^ ^46^, ^47^, ^48^ ^]^ Consistently, the upregulation of CCI‐induced inflammatory factors (*Tnf, Il6, Il1b*) and astrocyte‐related mediators (*Ccl2 and Cxcl1*) was reversed by AQP4 inhibition (Figure 4D1–D5). Notably, these gene expressions were also reversed by NFAT5 inhibitor administration (Figure S5C1–C5, Supporting Information), suggesting a link between NFAT5 and AQP4 in the regulation of neuroinflammation. Since NFAT5 directly regulates *Aqp4* transcription (Figure 3), these results suggested that targeting the NFAT5‐AQP4 signaling pathway modulates neuropathic pain by alleviating astrocyte swelling and neuroinflammation.

### 2.5 AURKB Promotes NFAT5 Nuclear Localization and Protein Stability

**Figure 5:**
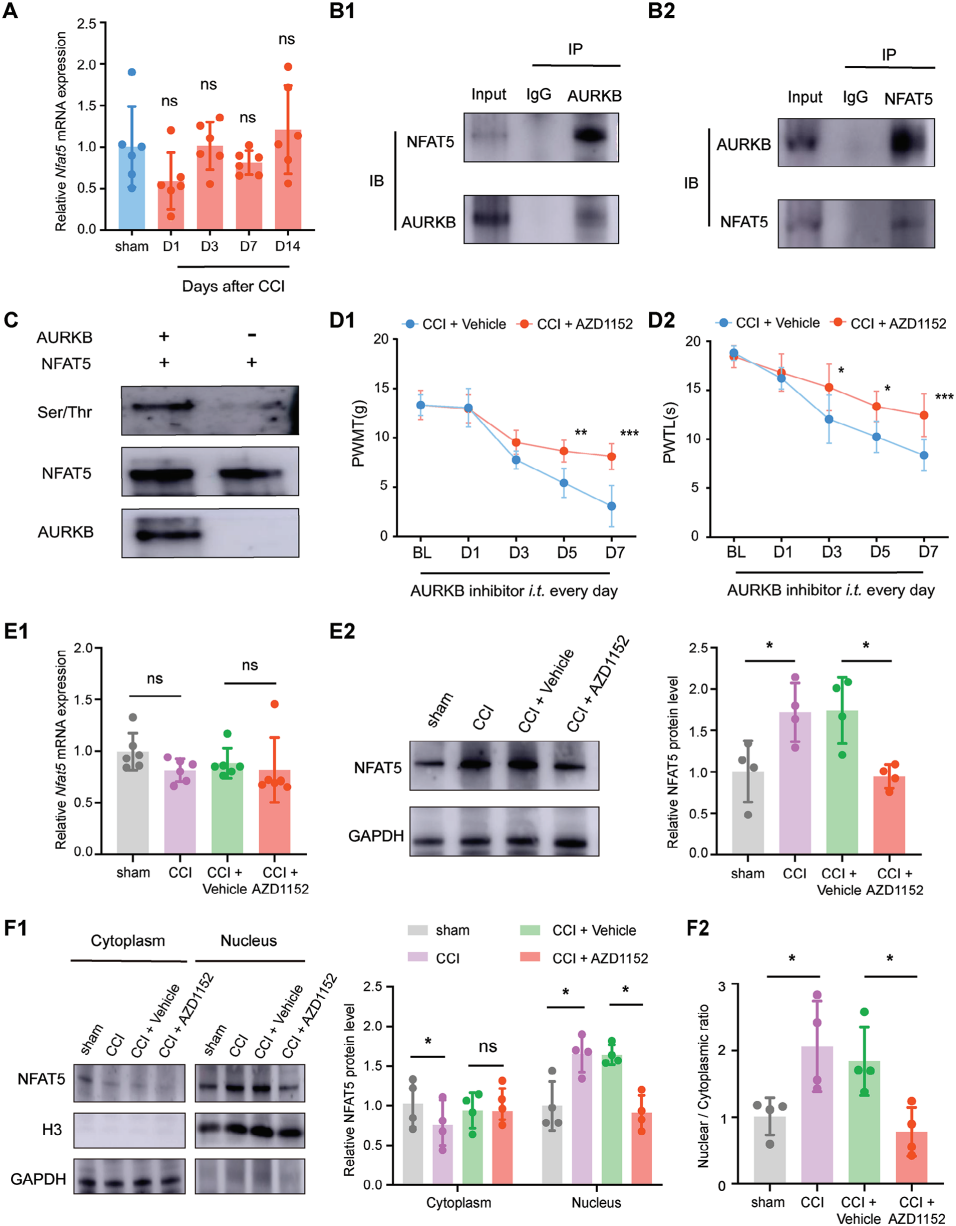
AURKB regulates NFAT5 phosphorylation and its protein level. A) Expression of *Nfat5* mRNA in the injured ipsilateral SDH after sham or CCI surgery. B1‐B2) Co‐immunoprecipitation of AURKB and NFAT5 in the SDH of rats after CCI modeling. C) Experimental validation of AURKB regulation of NFAT5 phosphorylation in vitro. D1‐D2) Behavioral tests of PWMT and PWTL after daily *i.t*. administration of AURKB inhibitor AZD1152 over 7 days post‐CCI modeling. *p < 0.05, **p < 0.01, ***p < 0.001, *n* = 6, two‐way ANOVA with repeated measures followed by Tukey's multiple comparison test. E1) *Nfat5* mRNA expression in the SDH after daily *i.t*. administration of AURKB inhibitor AZD1152 in CCI rats. E2) NFAT5 total protein expression in the SDH after daily *i.t*. administration of AURKB inhibitor AZD1152 in CCI rats. CCI versus sham, *p < 0.05, CCI + AZD1152 versus CCI + Vehicle, *p < 0.05, *n* = 4, one‐way ANOVA followed by Tukey's multiple comparison test. F1) Cytoplasm and nucleus NFAT5 protein levels after daily *i.t*. administration of AURKB inhibitor AZD1152 in the SDH of CCI rats. CCI versus sham, *p < 0.05, CCI + AZD1152 versus CCI + Vehicle, *p < 0.05, *n* = 4, one‐way ANOVA followed by Tukey's multiple comparison test. F2) Nuclear to cytoplasmic ratio of NFAT5 protein of F1, *p < 0.05, CCI + AZD1152 versus CCI + Vehicle, *p < 0.05, *n* = 4, one‐way ANOVA followed by Tukey's multiple comparison test.

Next, we seek to explore the mechanism behind the upregulation of NFAT5 after CCI surgery. Surprisingly, there was no significant change in *Nfat5* mRNA after CCI surgery (**Figure** **5**A), suggesting that CCI modulated NFAT5 protein levels through a post‐transcriptional mechanism. We previously demonstrated that AURKB, a serine/threonine protein kinase, plays a key role in neuropathic pain by regulating the phosphorylation of downstream substrates.^[^ ^49^ ^]^ Ingenuity Pathway Analysis (IPA) based on transcriptomes of AURKB knockdown CCI rats predicted that AURKB mediates inflammation and pain‐related genes through transcription factor NFAT5 (Table S6, Supporting Information). Thus, we hypothesized that AURKB might be the upstream regulator of NFAT5. Similar to NFAT5 localization (Figure S1A1–A2, Supporting Information), AURKB was also co‐labeled with the neuron, astrocyte, and microglia (Figure S6A, Supporting Information). Importantly, AURKB was widely co‐expressed with NFAT5 in the SDH of CCI rats (Figure S6B, Supporting Information). Moreover, the co‐immunoprecipitation (Co‐IP) experiment demonstrated an interaction between NFAT5 and AURKB in the SDH of CCI rats (Figure 5B1–B2), and in vitro phosphorylation assays confirmed AURKB's role in promoting the NFAT5 phosphorylation (Figure 5C). In addition, administration of AURKB specific inhibitor AZD1152 alleviated neuropathic pain, and reduced the protein amount of NFAT5 without altering *Nfat5* mRNA expression in the SDH (Figure 5D1–E2). Thus, these data suggested that AURKB acts as a posttranscriptional regulator of NFAT5 accumulation in vivo. It is known that the phosphorylation of NFAT5 may affect its nuclear localization.^[^ ^50^, ^51^ ^]^ To investigate this, we examined NFAT5 localization expression and found that nuclear expression of NFAT5 in rat SDH was significantly increased after CCI. Conversely, continuous intrathecal administration of the AURKB inhibitor significantly inhibited the nuclear expression of NFAT5 (Figure 5F1–F2). These results demonstrated that AURKB promotes NFAT5 protein entry into the nucleus.

Given that phosphorylation can modulate protein localization and protein stability,^[^ ^52^, ^53^ ^]^ we examined whether AURKB is required to maintain the stability of NFAT5 protein by reducing the degradation of NFAT5 protein in HEK293T cells, a classic human cell line for stability assay.^[^ ^54^ ^]^ Initially confirming the conservation of AURKB‐dependent regulation on NFAT5 in HEK293T cells, we observed that overexpression of AURKB increased NFAT5 accumulation, a trend reversed by AZD1152 administration (Figure S7A, Supporting Information). Subsequently, using the translation inhibitor cycloheximide (CHX) to induce NFAT5 protein turnover, we found that NFAT5 was gradually degraded over time. However, in HEK293T cells overexpressing AURKB, the NFAT5 protein half‐life significantly increased, an effect reversed by further administration of the AURKB inhibitor AZD1152 (Figure S7B1–B3, Supporting Information). Moreover, the proteasome inhibitor MG132 eliminated the differences in NFAT5 protein degradation levels caused by CHX (Figure S7C1–C2, Supporting Information). Collectively, these experimental results proved that AURKB enhances the stability of NFAT5 protein.

### 2.6 AURKB Regulates the NFAT5‐AQP4 Signaling Pathway in Astrocytes

**Figure 6:**
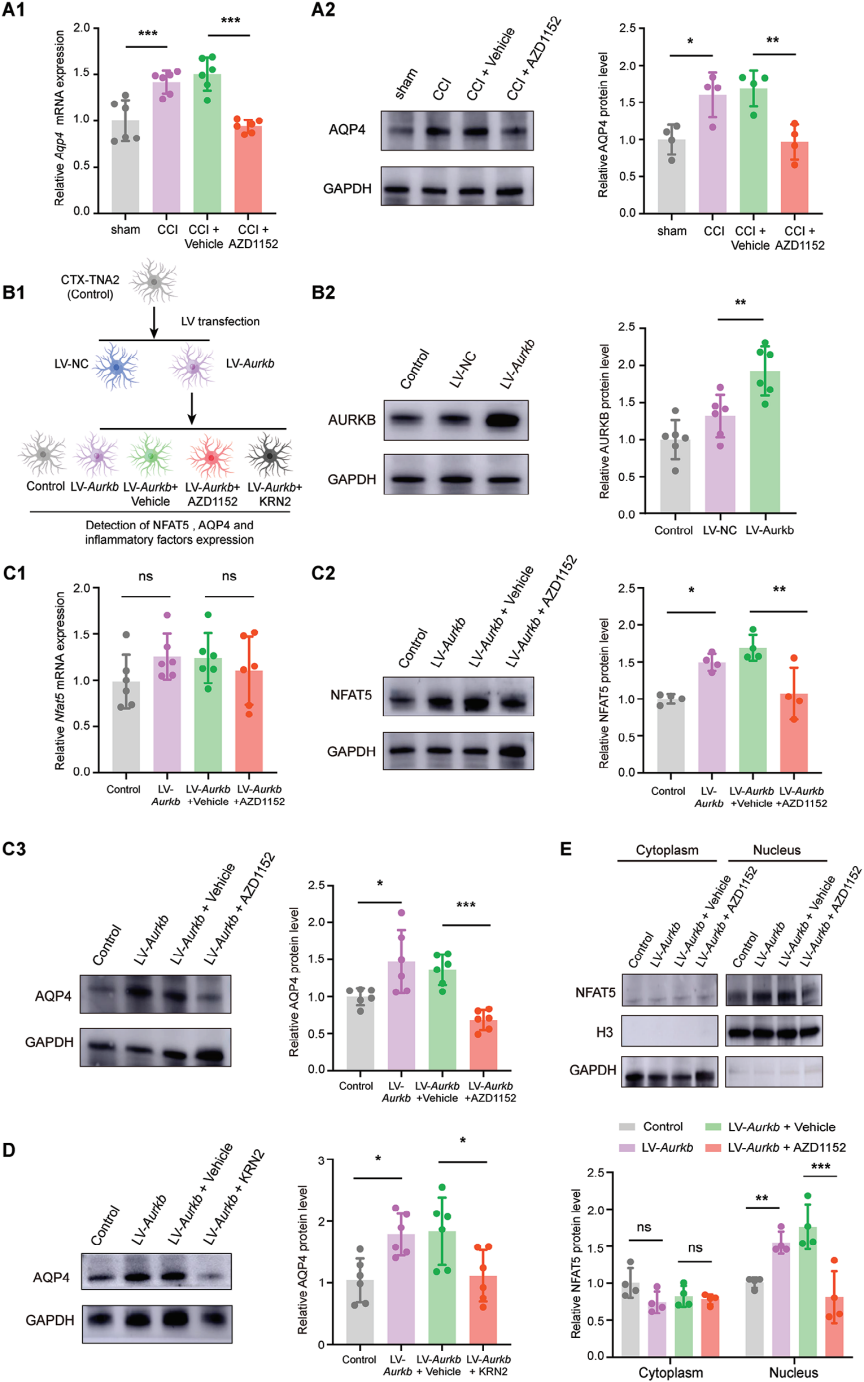
AURKB regulates NFAT5‐AQP4 signaling pathway in astrocytes. A1) *Aqp4* mRNA expression in the SDH of CCI rats after daily *i.t*. administration of AURKB inhibitor AZD1152. CCI versus sham, ***p < 0.001, CCI+ AZD1152 versus CCI+ Vehicle, ***p < 0.001, *n* = 6, one‐way ANOVA followed by Tukey's multiple comparison test. A2) AQP4 protein expression in the SDH of CCI rats after daily *i.t*. administration of AURKB inhibitor AZD1152. CCI versus sham, *p < 0.05, CCI + AZD1152 versus CCI + Vehicle, **p < 0.01, *n* = 4, one‐way ANOVA followed by Tukey's multiple comparison test. B1) Experimental procedure involved the overexpression of AURKB in CTX‐TNA2 cell line, followed by subsequent administration of the AURKB inhibitor AZD1152 or NFAT5 inhibitor KRN2. B2) The AURKB protein level in the LV‐*Aurkb* transfected CTX‐TNA2 cell line. LV‐*Aurkb* versus LV‐NC, **p < 0.01, *n* = 6, one‐way ANOVA followed by Tukey's multiple comparison test. C1‐C2) The *Nfat5* mRNA and protein levels in the LV‐*Aurkb* transfected CTX‐TNA2 cells with AURKB inhibitor AZD1152 treatment for 24 h. Control versus LV‐*Aurkb*, *p < 0.05, LV‐*Aurkb* + Vehicle versus LV‐*Aurkb* + AZD1152, **p < 0.01, *n* = 4, one‐way ANOVA followed by Tukey's multiple comparison test. C3) The AQP4 protein levels in the LV‐*Aurkb* transfected CTX‐TNA2 cells administration with AURKB inhibitor AZD1152 treatment for 24 h. Control versus LV‐*Aurkb*, *p<0.05, LV‐*Aurkb* + Vehicle versus LV‐*Aurkb* + AZD1152, ***p < 0.001, *n* = 6, one‐way ANOVA followed by Tukey's multiple comparison test. D) The AQP4 protein levels in the LV‐*Aurkb* transfected CTX‐TNA2 cells administration with NFAT5 inhibitor KRN2 treatment for 24 h. Control versus LV‐*Aurkb*, *p<0.05, LV‐*Aurkb* + Vehicle versus LV‐*Aurkb* + KRN2, *p < 0.05, *n* = 6, one‐way ANOVA followed by Tukey's multiple comparison test. E) NFAT5 protein level in cytoplasm and nucleus of AURKB overexpressed CTX‐TNA2 cells, followed with administered AURKB inhibitor AZD1152. Control versus LV‐*Aurkb*, **p<0.01, LV‐*Aurkb* + Vehicle versus LV‐*Aurkb* + AZD1152, ***p < 0.001, *n* = 4, one‐way ANOVA followed by Tukey's multiple comparison test.

**Figure 7:**
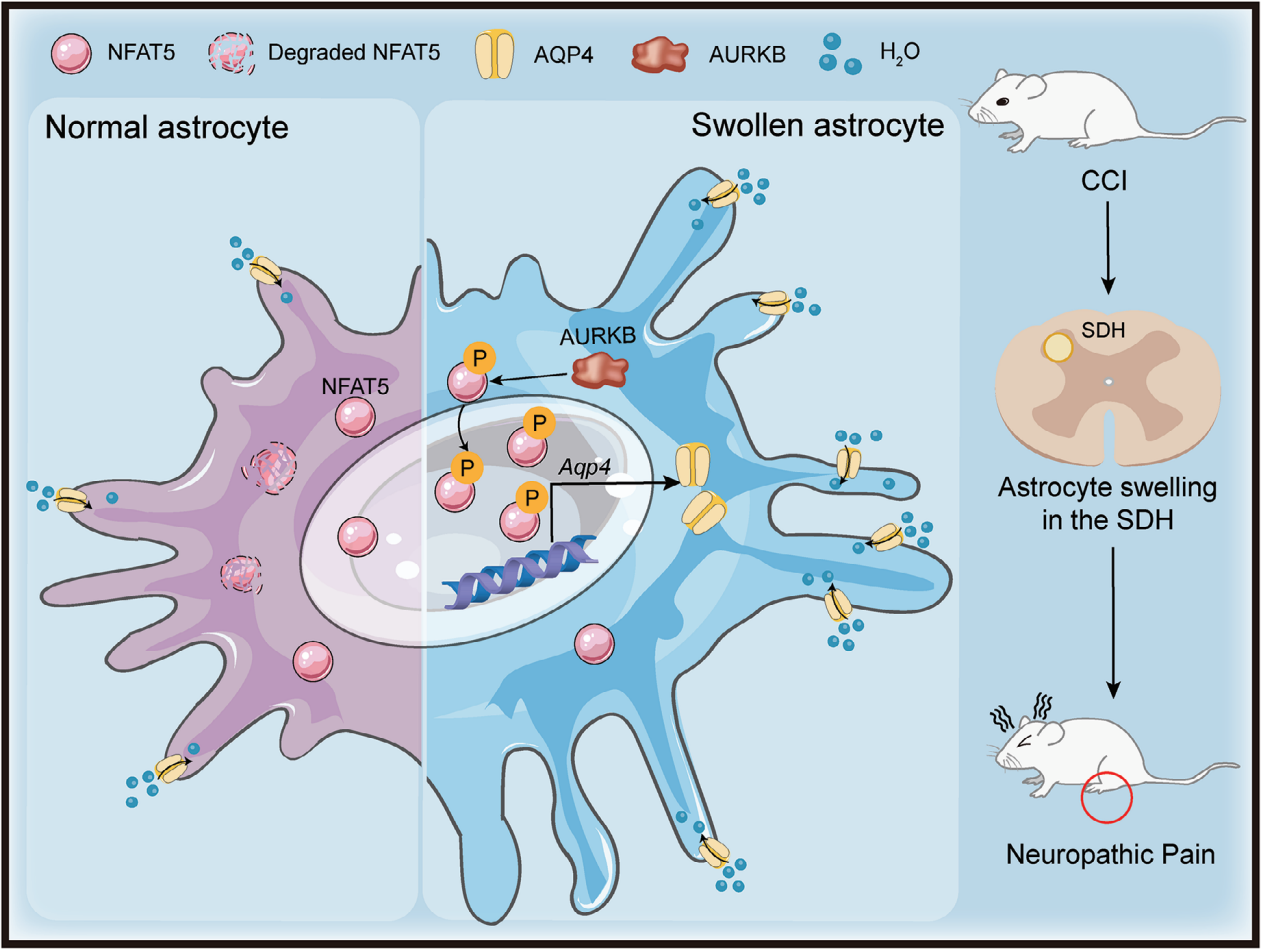
Schematic diagram of the NFAT5 signaling pathway regulation in SDH astrocytes during neuropathic pain. Peripheral nerve injury induces morphological changes in SDH astrocytes, characterized by significant cell swelling, a prominent pathological feature in the context of neuropathic pain. The NFAT5 signaling pathway plays a crucial role in astrocyte swelling after peripheral nerve injury. AURKB mediates NFAT5 phosphorylation, promotes NFAT5 translocation into the nucleus and protects it from degradation. The nuclear NFAT5 further stimulates the transcription and expression of AQP4, which subsequently leads to astrocyte swelling and contributes to the development of neuropathic pain.

To test whether AURKB regulated the NFAT5‐AQP4 pathway, we checked the *Aqp4* expression in the RNA‐seq data of AURKB knockdown CCI rats, finding that the increased *Aqp4* was reversed by AURKB knockdown (Figure S6C, Supporting Information). Furthermore, administration of AURKB inhibitor AZD1152 significantly reduced the mRNA and protein expression level of AQP4 after CCI surgery (**Figure** **6**A1–A2), indicating that AURKB regulated the NFAT5‐AQP4 pathway in vivo. We further validated this circuit using the rat astrocyte cell line CTX‐TNA2 (Figure 6B1). After confirming CTX‐TNA2 cell purity by GFAP immunostaining (Figure S8A1, Supporting Information), enhanced NFAT5 protein expression upon overexpression of AURKB was observed in CTX‐TNA2 cells (Figure 6B2; Figure S8A2–A3, Supporting Information). Conversely, AURKB inhibition significantly reversed the increase in NFAT5 protein but had no effect on NFAT5 mRNA expression (Figure 6C1–C2), consistently with in vivo data (Figure 5A,E1‐E2). Similarly, AQP4 was dramatically increased in AURKB overexpressing cells, and its expression was significantly reversed in the presence of the AURKB AZD1152 inhibitor (Figure 6C3). Moreover, the increased expression of AQP4 protein caused by AURKB overexpressing could be reversed by the NFAT5 inhibitor KRN2 (Figure 6D). Overexpression of AURKB in CTX‐TNA2 cells also caused elevated levels of NFAT5 protein in the nucleus, which was reversed by AZD1152 administration (Figure 6E). Additionally, overexpression of AURKB resulted in increased inflammatory factor expression in CTX‐TNA2 cells, an effect reversed by inhibition of AQP4 (Figure S8B1–B3, Supporting Information), consistent with the in vivo results (Figure 4D1–D5; Figure S5C1–C5, Supporting Information). These experiments collectively demonstrate that AURKB positively regulates NFAT5 and its downstream target AQP4 in astrocytes, suggesting that the AURKB‐NFAT5‐AQP4 pathway could mediate neuroinflammation via astrocytes (**Figure** **7**).

## 3 Discussion

Peripheral nerve injury is widely recognized to activate astrocytes in the SDH, contributing to the development of neuropathic pain. However, the specific role of astrocyte swelling, induced by water influx, in this process remains unclear. Our current study demonstrates that chronic neuropathic pain triggers astrocyte swelling, with NFAT5 identified as a key regulator of this process. Inhibition of NFAT5 signaling attenuated the astrocyte swelling in the SDH, providing relief from neuropathic pain. Notably, NFAT5 appears to promote astrocyte swelling through transcriptional regulation of AQP4, the non‐selective, bidirectional water channels that facilitate the passive diffusion of water across cellular membranes. These findings offer a novel explanation for how NFAT5 signaling mediates morphological changes in astrocytes under neuropathic pain conditions caused by peripheral nerve injury. Consequently, NFAT5 signaling emerges as a potential therapeutic target for treating neuropathic pain induced by peripheral nerve injury.

It is established that the initial brain injury can lead to brain tissue swelling, with astrocyte swelling being the primary cause of brain edema.^[^ ^18^, ^20^ ^]^ However, limited studies have explored cellular swelling in the CNS initiated by peripheral nerve injury. Taking advantage of immunofluorescence confocal 3D reconstruction and TEM ultrastructural technologies, this study demonstrated that peripheral nerve injury leads to cellular morphological changes in the SDH. This includes reduced processes length and increased cell volume in microglia, and increased processes length and cell volume in astrocytes. Strikingly, CCI induces prominent swelling in astrocytes but not in microglia of the SDH. Astrocytes, compared to neurons and microglia, express a higher abundance of ion and water channel proteins. Alterations in the expression or function of these channel proteins can disrupt fluid imbalance within astrocytes, rendering them more susceptible to swelling changes.^[^ ^55^, ^56^ ^]^ Although NFAT5 is ubiquitously expressed in various cell types in the SDH, it specifically promotes the swelling in the astrocytes, possibly by regulating the expression of AQP4 which is predominantly expressed in astrocytes. AQP4, the master regulator of the water channel, tightly controls water fluxes into and out of the cell.^[^ ^45^ ^]^ Increased AQP4 expression leading to excessive water influx is implicated in astrocyte swelling in pathological conditions such as cerebral edema and traumatic brain injury.^[^ ^57^, ^58^, ^59^ ^]^ Thus, peripheral nerve injury may activate NFAT5‐AQP4 signaling, culminating in astrocyte swelling in the SDH.

In the context of CNS injury models, astrocyte swelling is an important component of astrogliosis, accompanying the functional changes in reactive astrocytes, including the release of inflammatory mediators.^[^ ^20^ ^]^ This study also observed that inhibition of NFAT5 and AQP4 significantly inhibits inflammatory factors such as *Tnf*, *Il6*, and *Il1b*, as well as chemokines such as *Ccl2* and *Cxcl1*, closely associated with reactive astrocytes responses.^[^ ^44^, ^60^ ^]^ This might be particularly important since reactive astrocytes in the SDH have been identified as one of the predominant factors that contribute to the development and maintenance of neuropathic pain following peripheral nerve injury.^[^ ^6^, ^61^ ^]^ Thus, astrocyte‐specific targeting NFAT5 and its downstream target AQP4 could alleviate the peripheral nerve injury induced by neuropathic pain. However, this study does not discount the function of NFAT5 in non‐astrocytes, given its ubiquitously expressed in various cell types in the SDH. Another report also suggests that NFAT5 might regulate neuropathic pain through neurons and microglia in the SDH.^[^ ^62^ ^]^ We also confirmed the enhanced enrichment of NFAT5 on the promoters of Reg3a and Ifi47 after CCI surgery (Figure.S5 G1‐G2, Supporting Information). In addition, NFAT5 is reported to regulate gene transcription of several inflammatory factors such as *Il1b* and *Tnf* in macrophages.^[^ ^29^, ^63^ ^]^ We also observed an enhanced enrichment of NFAT5 in the inflammation cytokine gene *Il6* (Figure 3B; Table S5, Supporting Information). Thus, general targeting of NFAT5 has a more pronounced effect on neuropathic pain than astrocyte‐specific targeting, indicating that the contribution of NFAT5 to neuropathic pain may extend beyond astrocytes. Future studies are needed to decipher the cell‐specific functions of NFAT5 in neuropathic pain.

An intriguing aspect of this study is the expression of NFAT5 during neuropathic pain. The increase in protein levels without a corresponding increase in mRNA transcription after CCI surgery underscores the importance of post‐transcriptional regulation of NFAT5. NFAT5 localization and function are highly mediated by different kinases in a context‐dependent manner.^[^ ^50^, ^51^ ^]^ This study reveals that AURKB tightly regulates NFAT5 signaling. AURKB, a serine‐threonine kinase implicated in the pathogenesis of neuropathic pain,^[^ ^49^ ^]^ phosphorylates NFAT5, facilitating its nuclear accumulation by inhibiting degradation. This regulation by AURKB is consistent with previous reports showing that AURKB not only phosphorylates the substrate but also hinders the interaction between the substrate and E3 ubiquitin ligase, thereby reducing the subsequent ubiquitin‐mediated proteasomal degradation.^[^ ^64^ ^]^ Importantly, these findings suggest that the AURKB‐dependent regulation of NFAT5 is conserved in human cells. Considering the ongoing clinical trials of AURKB inhibitor,^[^ ^65^ ^]^ targeting AURKB could hold promise for clinical translation of the treatment of NFAT5 signaling–regulated astrocyte swelling and neuropathic pain. Open questions still remain, including the specific phosphorylation sites of NFAT5 catalyzed by AURKB, the potential involvement of other kinases in NFAT5 function in chronic pain, and the mechanisms through which phosphorylation influences its transactivation activity.

Given the consistent astrocyte activation during persistent pain conditions and the well‐established role of astrocytes in neuropathic pain, directing therapeutic interventions toward reactive astrocytes holds significant promise.^[^ ^10^ ^]^ This study sheds light on the crucial role of the NFAT5 pathway in mediating astrocyte swelling in the SDH induced by peripheral nerve injury. This swelling mechanism contributes to the morphological alteration in astrocytes, potentially fostering the development of astrogliosis and neuropathic pain. Specifically, NFAT5 orchestrates astrocyte swelling through the mediation of AQP4, and this NFAT5‐AQP4 pathway is subject to regulation by the upper stream kinase AURKB. Targeting the NAFT5 signaling emerges as an effective strategy for alleviating astrocyte swelling and, consequently, treating neuropathic pain induced by peripheral nerve injury.

## 4 Experimental Section

### Animals

Adult male and female Sprague–Dawley (SD) rats were obtained from Hunan SJA Laboratory Animal. The rats were kept in a controlled environment with temperature maintained at 25–28°C and humidity at 50% −60%. They followed a 12‐hour light and darkness cycle, with ad‐libitum access to food and water. All procedures were approved by the Institutional Ethics Committee of Xiangya Hospital, Central South University, and adhered to the National Institutes of Health Guidelines for Laboratory Animal Care and Ethical Guidelines.

### Rat Model of CCI

The CCI model was established following the method developed by Bennett and Xie.^[^ ^66^ ^]^ After anesthetizing the rats with isoflurane, the sciatic nerve of the left hind limb was exposed. Four square knots were tied on the main nerve trunk using a 4‐0 chromium enteric line at 1 mm intervals. The knot intensity was adjusted to cause a slight tremor in the peripheral muscle group while ensuring mild compression of the sciatic nerve membrane without affecting blood supply. For sham surgery, the sciatic nerve was exposed without ligation.

### Behavioral Test

The pain behavioral tests were performed in a double‐blind manner as described below.^[^ ^67^ ^]^

*PWMT*: VonFrey filaments (North Coast Medical) were used to measure ipsilateral PWMT in rats. In brief, rats were placed on a behavioral measurement rack (metal mesh) and separated by a transparent plexiglass box (22 cm × 12 cm × 22 cm). After the rats entered a quiet state, VonFrey filaments were used to vertically stimulate the rats' middle plantar of the left hind limb, starting from 0.4 g and reaching a maximum of 15 g. PWMT of rats was calculated by the UP AND DOWN method.^[^ ^67^ ^]^

*PWTL*: A thermal pain test instrument (Ugo Basile) measured ipsilateral PWTL. Briefly, rats were isolated to a plastic partition on the surface of a 2 mm thick thermal glass plate for 30 minutes. The radiation source center of the thermal pain test instrument was used to measure the left central plantar of the rats. Repeated 3 times with an interval of 5 min and the mean latency period of each measurement was recorded as PWTL. To prevent tissue damage in rats, the cut‐off value of the thermal pain test instrument was no more than 30 s.

Spontaneous pain behavioral observation: Utilizing the method established in a prior study,^[^ ^68^ ^]^ animals were situated in transparent containers. The occurrences of licking and episodes of flinching/shaking/lifting involving the ipsilateral hind paw were tallied over a 60‐minute duration to assess spontaneous pain behaviors. Movements related to exploratory behavior, locomotion, and body adjustments were excluded. Each bout of licking was recorded as 2 points, and each bout of flinching/shaking/lifting was recorded as 1 point. The cumulative score for 60 min constituted the spontaneous pain score.

### Plasmids Construction and Virus Production

For NFAT5 non‐specific knockdown, a recombinant adeno‐associated virus (rAAV‐U6‐shRNA(*Nfat5*)‐CMV‐EGFP‐SV40; aav2/9) was used.^[^ ^69^ ^]^ For astrocytes‐specific knockdown of NFAT5, a recombinant adeno‐associated virus (rAAV‐GFaABC1D‐mCherry‐shRNA(*Nfat5*)‐WPREs; aav2/5) was employed according to previous studies.^[^ ^38^ ^]^ The shRNA sequence of rat *Nfat5* gene (NM_0 011 07425.1) was 5′‐GCGGCACAGTTTCAGACAAGA‐3′; the scrambled control sequence was 5′‐CCTAAGGTTAAGTCGCCCTCG‐3′. All AAVs were from BrainVTA and prepared in phosphate‐buffered saline (PBS) solution. The design knockdown and overexpressed vectors of rats' *Aurkb* gene were described as previous.^[^ ^49^ ^]^

### Intrathecal Delivery of Virus or Drugs

The method of intrathecal catheter implantation in rats was adopted from a previous study.^[^ ^49^ ^]^ After the rats were anesthetized, the lumbar intervertebral space was fully exposed, and the puncture was performed at the L4‐5 space. Then the PE10 catheter was placed into the subarachnoid space. Once monitoring the slow outflow of cerebrospinal fluid, the tip of the catheter was closed and properly fixed. The lidocaine test was performed on the third day after the intrathecal catheter implantation by injecting 10 µL lidocaine into the catheter. If the bilateral hind limbs paralysis reaction occurred within 1 min after injection, the intrathecal catheter implantation was successful and ready to use for subsequent administration of virus and inhibitor drugs. Otherwise, the lidocaine test was negative, and such rats were excluded from the subsequent experiment.

The AAV‐shRNA (AAV‐sh*Nfat5* or AAV‐sh*Aurkb*) or LV‐*Aurkb* as well as their corresponding negative control (AAV‐NC or LV‐NC) were delivered intrathecally 2 weeks before sham or CCI surgery. AURKB inhibitor (AZD1152, 50 µg kg^−1^, Selleck), NFAT5 inhibitor (KRN2, 5 µg kg^−1^ or 20 µg kg^−1^, MCE), or AQP4 inhibitor (TGN‐020, 20 µg kg^−1^, Selleck) were intrathecally injected into CCI rats. The vehicle animals were administered with the same volume of 1% DMSO in the saline solution.

### AAV Intraspinal Injection

Intraspinal injection of AAV in rats followed a previous protocol as described.^[^ ^70^ ^]^ After anesthetization, the skin and muscle were incised to fully expose the T12‐L1 vertebral plate, and a portion of the plate was carefully removed to expose the underlying spinal cord. Then the AAV (1 µl) was injected into the rat SDH using a stereotaxic and microinjection device. The injection location was 0.8 mm to the left at the midline of the spinal cord, and the needle tip was inserted at a depth of 0.6 mm. These rats were recovered for 2 weeks for subsequent experiments.

### Western Blot and Co‐IP

Total protein was extracted with RIPA lysate, and cytoplasmic/nuclear proteins were extracted using the Nuclear and Cytoplasmic Protein Extraction Kit (TransGen Biotech). After determining the concentration by the BCA method, proteins were heated at 99°C for 10 min, subjected to SDS‐PAGE gel electrophoresis, and transferred to the PDVF membrane. Immunoblots were carried out with primary antibodies, including anti‐NFAT5 (Abcam, ab3446), anti‐AURKB (Abcam, ab2254), anti‐Phospho‐Ser/Thr (Abcam, ab17464), anti‐AQP4 (Abcam, ab2254), anti‐GAPDH (ZENBIO, 20030–67E4), anti‐Histone 3 (CST, 4499s).

Co‐IP assay was performed using a Direct Magnetic IP/CO‐IP Kit (Thermo Fisher Scientific, 88 824). Briefly, rat SDH tissue was lysed with IP lysis buffer supplied with protease and phosphatase inhibitors, and then incubated with the primary antibodies: anti‐NFAT5 (Novus Biologicals, NB1203446), or anti‐AURKB (Abcam, ab2254), or anti‐IgG (Proteintech, B900610). Magnetic beads (Thermo Fisher Scientific, 88 824) were used to pull down the corresponding protein complex. After eluted the proteins from the beads, and western blot was used to identify the specific protein.

### Real‐Time Quantitative Polymerase Chain Reaction (RT‐qPCR)

Total RNA was extracted from tissues and cells using a total RNA extraction kit (Transgen), and then reverse‐transcribed into cDNA using the First‐Strand cDNA Reverse Transcription SuperMix kit (Transgen). SYBR Green qPCR SuperMix (Transgen) was used for RT‐qPCR on the ABI QuantStudio 5 instrument. Then Ct values of target genes and reference genes were used for analysis by the 2^−ΔΔCt^ method. The primer sequences are listed in Table S1 (Supporting Information).

### Cell Culture and Transfection

HEK293T cells and CTX‐TNA2 cells were purchased from Procell Life Science & Technology. Cells were cultured with DMEM medium with 10% (v/v) fetal bovine serum, and 1% (v/v) penicillin and streptomycin in the 37°C incubator with 5% CO_2_. Cells were transfected with LV‐*Aurkb* or LV‐NC virus using an auxiliary transfection reagent (BrainVTA). After 72 h, the cells were collected for fluorescence detection, RNA isolation or western blot. AURKB inhibitor (AZD1152, 50 µM, Selleck), NFAT5 inhibitor (KRN2, 50 µm, MCE) and AQP4 inhibitor (TGN‐020, 20 µM, Selleck) were added at 72 h post‐transfection.

### Calcein AM Permeation Assay

Cells were incubated with Calcein AM (5 µm, Beyotime) at 37°C for 30 min. Calcein AM can easily penetrate the living cell membrane and is hydrolyzed to Calcein by intracellular esterase, Calcein remains inside the cell and emits a strong green fluorescence. After the incubation, each group of cells was changed to a normal medium and incubated for another 30 min under protection from light to ensure that the intracellular esterase fully hydrolyzed Calcein AM. Then the swelling of cells was observed under the microscope.

### Immunofluorescence and Microscopy

Immunofluorescence experiments were performed on rat spinal cord sections and rat astrocyte CTX‐TNA2 cell line. Frozen sections of rat spinal cord and slides of CTX‐TNA2 cells were prepared and blocked using QuickBlock Immunostaining block solution (Beyotime). After incubated with primary antibodies: anti‐NeuN (Novus Biologicals, NB1‐92693), anti‐IBA1 (Wako, 011–27991), anti‐GFAP (CST, 3670S), anti‐NFAT5 (Abcam, ab3446), anti‐AURKB (Abcam, ab2254), or anti‐AQP4 (Abcam, ab2254); the corresponding secondary fluorescent antibodies were used to release the signals. The slides were mounted with the permanent mounting medium and imaged under the Leica DMI4000 fluorescence microscope with the DFC365FX camera (Leica).

### 3D Cell Morphology Analysis

3D cell morphology was analyzed using confocal microscopy as described in previous studies.^[^ ^71^, ^72^ ^]^ SDH astrocytes and microglia were labeled with GFAP and IBA1, respectively. Using a Zeiss confocal microscope (10×, 2048 × 2048 pixels, 8‐bit depth) with Z‐axis spacing set to 2 µm, high‐magnification was set to (40×, 2048 × 2048 pixels, 8‐bit depth, Z‐axis spacing = 1 µm), and successive images were captured until the cell morphology was complete in the field of view. Z‐stack pictures were exported, and ImageJ with AnalyzeSkeleton (2D/3D) and 3D Objects Counter plugins was employed to calculate the average processes length and volume of glial cells.

*Sholl Analysis*: Following the methods of a previous study,^[^ ^34^ ^]^ the photographed high‐resolution astrocytes were projected on the Z‐axis at maximum density to collapse Z‐stacks, and adaptive threshold filtering was applied to create the binary mask. After binarization, concentric circles of increasing radius (from 5 µm to 50 µm in steps of 5 µm) were created from the center of the astrocyte soma using ImageJ's Sholl analysis plugin, and the number of intersections of cell processes with these concentric circles at different distances was counted.

*Estimation of Processes Volume Fraction*: Astrocyte processes volume fraction was estimated as previously described.^[^ ^35^ ^]^ High‐resolution images containing astrocyte soma were selected. To reduce selection bias, 12 radial lines were drawn at the 30° angle from the center of the soma. Fluorescence profiles along these lines were obtained and the average fluorescence spectrum for each cell was calculated. The fluorescence spectrum values were divided by the peak fluorescence of the soma to obtain an estimate of the volume fraction of the astrocyte processes at different distances. The final astrocyte processes volume fraction for each group was calculated from the average volume fraction at different distances (from 5 to 40 µm).

### Transmission Electron Microscopy

Rats were anesthetized with 3% sodium pentobarbital and sequentially perfused with PBS (100 mL) and 4% paraformaldehyde solution (100 mL). The lumbar extension of the spinal cord was then dissected and cut into 1 × 1 × 2 mm tissue blocks. The tissues were then immersed in a mixture of 2.5% glutaraldehyde and 2.5% paraformaldehyde solution at 4°C overnight. After dehydration and permeation, the tissues were immersed in linoleate overnight, then sectioned and examined by TEM for morphological and structural changes associated with astrocytes and microglia at SDH. The somatic cytoplasm size of astrocytes was assessed using ImageJ software with the following formula: somatic cytoplasm size = overall cell area – area of the nucleus as described.^[^ ^73^, ^74^ ^]^ All data were analyzed and quantified by in blinded manner.

### In Vitro Protein Kinase Assay

Recombinant AURKB protein (0.1 µg, Abnova) and NFAT5 protein (1 µg, Abnova), ATP and kinase buffer (Cell Signaling Technologies) were co‐incubated for 30 min at 30°C. 4X SDS loading buffer were used to stop the reaction, and the reaction mixture was then boiled for 10 min at 95°C before being separated by SDS‐PAGE gel electrophoresis.

### Time‐Course Analysis of NFAT5 Degradation

Normal HEK293T cells and AZD1152‐treated *Aurkb* overexpressing HEK293T cells were treated with the protein synthesis inhibitor cycloheximide (CHX, 50 mg mL^−1^) and harvested at specific time points (0, 30, 60, 90, 120 min). Proteins were collected and immunoblotted. Additionally, cells were treated with the proteasome inhibitor MG132 (10 µm) for 3 h before CHX treatment.

### RNA‐Seq and Data Analysis

RNA of CCI and sham group rats SDH samples were purified with a RNeasy mini kit (Qiagen) according to the manufacturer's instructions. The RNA sequencing libraries were constructed and sequenced by Shanghai Biotechnology Company. rRNA reads, sequencing adapters, short fragments, and other low‐quality reads were removed. Clean reads were mapped to the rn6 reference genome using the HISAT2 (version:2.0.4). Gene expression was standardized to fragments per kilobase of per million mapped readings (FPKM). Gene ontology (GO) analysis and Kyoto Encyclopedia of Genes and Genomes (KEGG) analysis were performed for function annotation of differential genes.^[^ ^75^, ^76^ ^]^ IPA analysis was performed with IPA software (Qiagen, http://www.ingenuity.com/products/ipa).^[^ ^77^ ^]^

### ChIP‐Seq and ChIP‐qPCR

ChIP assays were performed using the ChIP‐kit (Millipore) according to the manufacturer's instructions. Briefly, the sham and CCI group SDH tissues were collected and cross‐linked with 1% formaldehyde. These cross‐linked cells were lysis and chromatin DNA was sonicated into 200–700 bp. After the reversal of protein‐DNA cross‐links, the chromatin DNA precipitated by anti‐NFAT5 (Novus Biologicals, NB120‐3446) was purified using the PureLink kit (Invitrogen). ChIP‐seq libraries were performed and analyzed with the assistance of Crystal Energy Biotechnology. NFAT5 ChIP‐seq raw reads were quality‐checked with FastQC (version v0.11.5) and then filtered to map to the rn6 reference genome. Using the commonly used short sequence fast alignment program Bowtie, the original sequences of the sample were analyzed by unique mapping with the reference genome. Peaks were called with MACS2. Enriched binding peaks of NFAT5 were generated after filtering through the control group. The distance ≤ +5 kb from the transcription start site was defined as the promoter region.^[^ ^78^ ^]^

For the ChIP‐q‐PCR assay, primers explicitly targeted to genes promoter region were designed as, *Aqp4*: forward primer 5′‐CACAAAGTGCCAGCAACACT‐3′, reverse primer 5′‐TGCCCAATAAAATCCACCTC‐3′, *Reg3a*: forward primer 5′‐TCCTTTGTCGATGGGAGCTT‐3′, reverse primer 5′‐ TGGTATCCAAGTCCAGAG

CA‐3′, *Ifi47*: forward primer 5′‐ TGCCTTTTGATGCCAGTTGT‐3′, reverse primer 5′‐ TTTACTCATGGGCCGACGAT‐3′. Samples were run in triplicate, and data from NFAT5 IP and IgG IP were presented as enrichment relative to the input DNA.

### Statistical Analysis

Data were presented as mean ± SD. The Shapiro‐Wilk test was used for data distribution. Two‐tailed unpaired Student's t‐test compared two groups, and one‐way or two‐way ANOVA followed by Tukey's multiple comparison test compared multiple groups. Statistical significance was set at *p* < 0.05 using GraphPad Prism 7.0 software.

## Conflict of Interest

The authors declare no conflict of interest.

## Author Contributions

L.H. and S.M. contributed equally to this work. C.H. and Q.G. conceived the project. C.H. and S.M. designed the experiments. L.H., Z.D., Z.H., Y.Z., C.X., K.Z., and Q.D. performed the experiments. L.H. and S.M. interpreted the data. L.H. and S.M. wrote the manuscript with input from C.H. and W.H. All authors read and approved the final manuscript.

## Supporting information

Supporting Information

## Data Availability

The data that support the findings of this study are available from the corresponding author upon reasonable request.
