# Supplementary material for: Inhibition of NFAT5‐Dependent Astrocyte Swelling Alleviates Neuropathic Pain
Source: Adv Sci (Weinh). 2024 Jan 9;11(11):2302916. doi: 10.1002/advs.202302916 (PMC10953562; doi:10.1002/advs.202302916)
Supplement: Supplementary file 1 — Supporting Information [file ADVS-11-2302916-s001.pdf]

ADVANCED SCIENCE
Open Access

Supporting Information

for *Adv. Sci.*, DOI 10.1002/advs.202302916

Inhibition of NFAT5-Dependent Astrocyte Swelling Alleviates Neuropathic Pain

*Liqiong He, Shengyun Ma, Zijin Ding, Zhifeng Huang, Yu Zhang, Caiyun Xi, Kailu Zou, Qingwei Deng, Wendy Jia Men Huang, Qulian Guo and Changsheng Huang**

Supplementary Materials

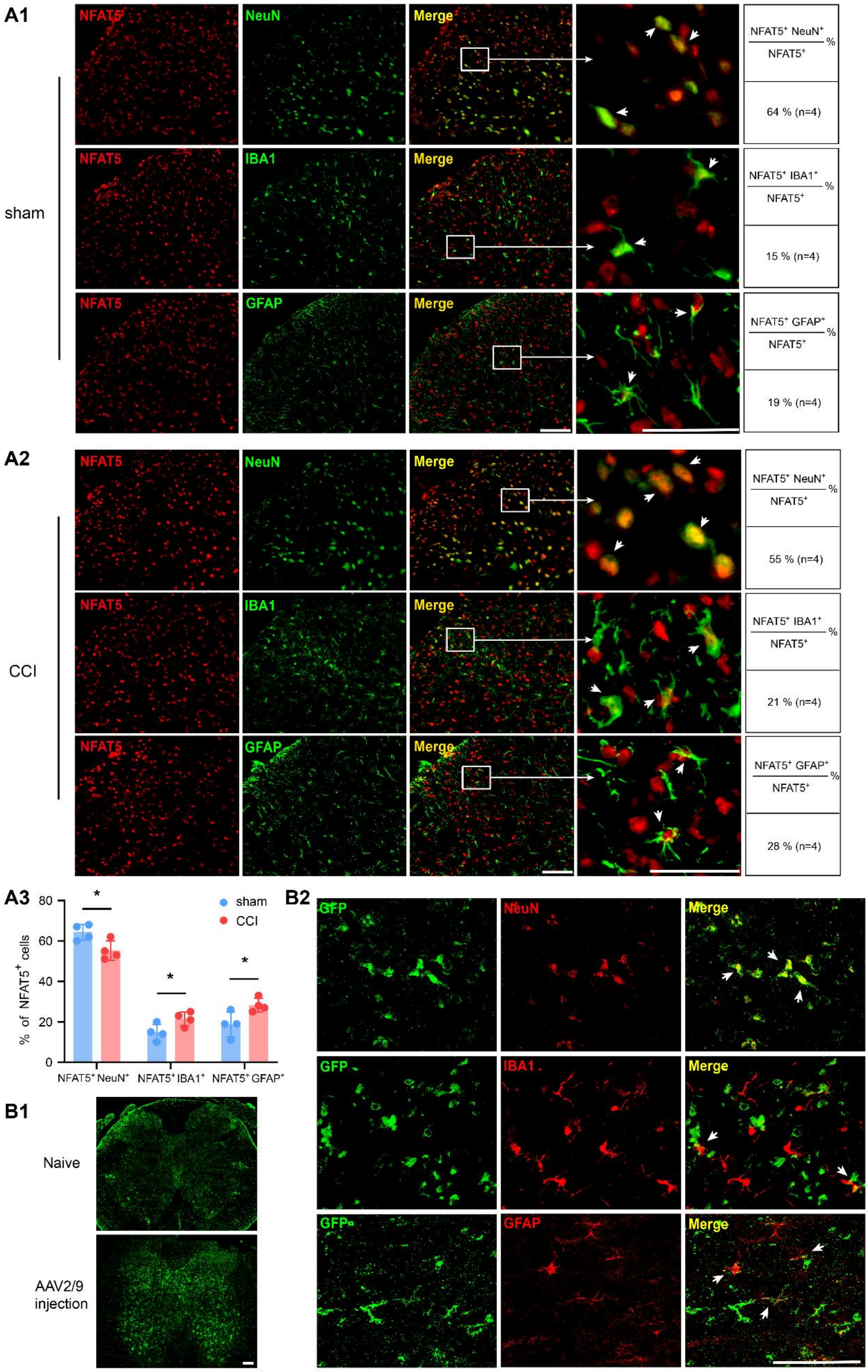

Figure. S1 NFAT5 distribution in SDH cells and fluorescent images after AAV2/9 transfection.

(A1-A2) Immunofluorescence double-labeling of NFAT5 (red) and indicated cell markers (green) in the SDH on day 7 after sham or CCI surgery. Quantification of cellular distribution was shown on the right (pooled from 4 rats/group). Scale bar = 100 µm. **(A3)** Statistics of NFAT5 distribution in SDH cells on day 7 after sham or CCI surgery. $*p < 0.05$, $n = 4$, two-tailed unpaired student's t-test. **(B1-B2)** GFP fluorescence images of spinal cord after NFAT5 non-specific AAV2/9 transfection and immunofluorescence double-labeled images of GFP (green) and various cell markers (red). Scale bar = 100 µm.

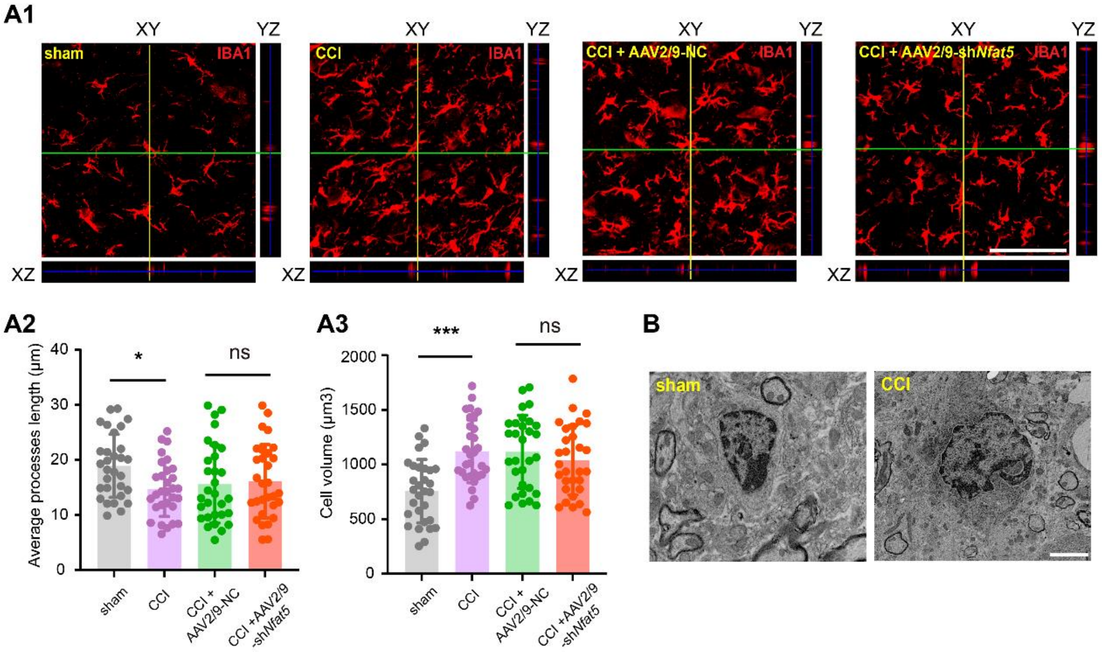

Figure. S2 Targeting NFAT5 does not impact microglia morphology.

(A1-A3) Representative 3D orthogonal confocal images (A1) and summarized morphological changes (A2-A3) of IBA1-stained microglia in indicated group of rats SDH. Scale bar = 100 μm. CCI vs sham, *p < 0.05, ***p < 0.001, n = 30 (3 rats per group, and 10 typical cells per rat were analyzed), one-way ANOVA followed by Tukey's multiple comparison test. **(B)** Representative transmission electron microscopy images of microglia in sham and CCI groups of rats SDH. Scale bar = 2 μm.

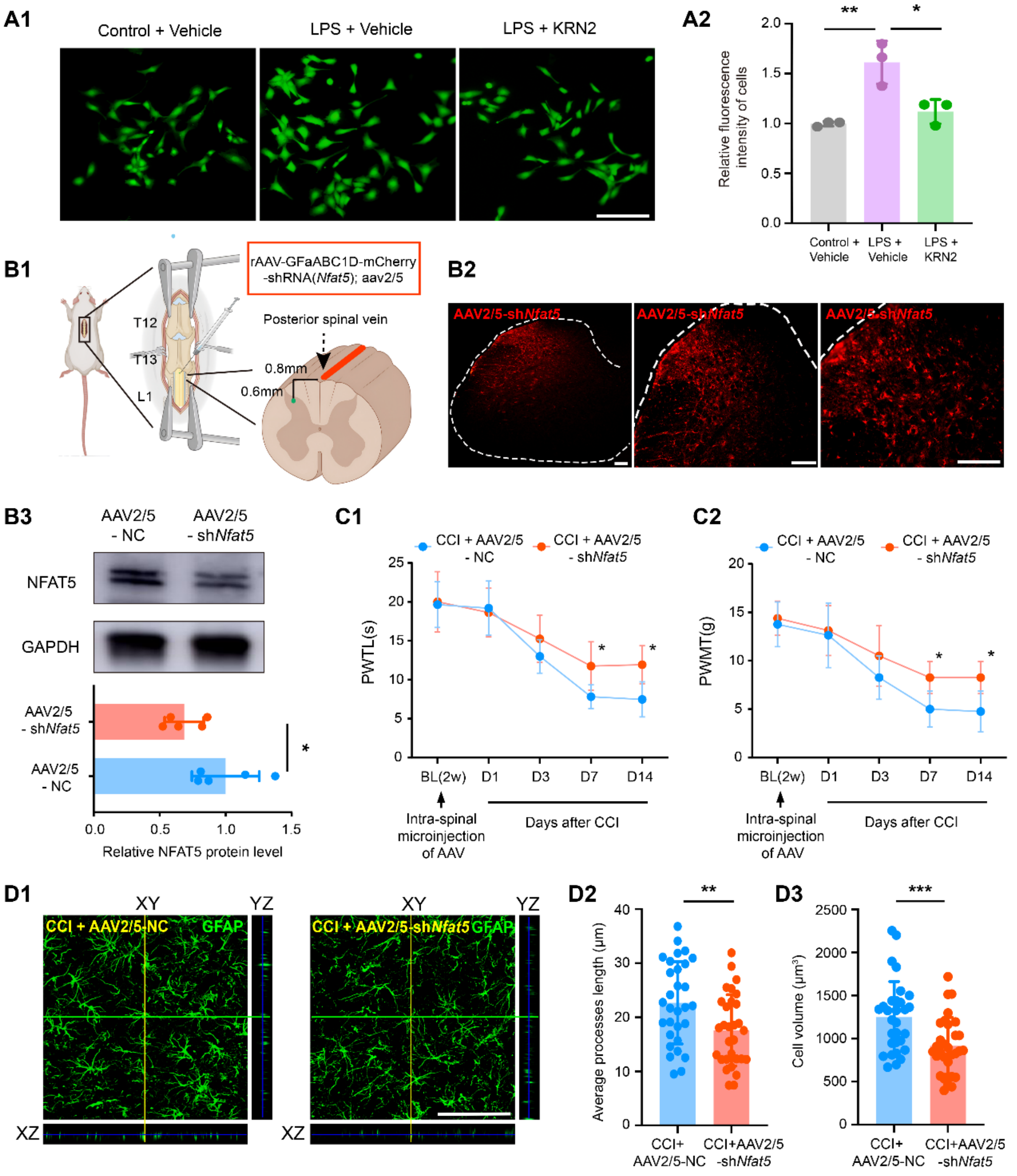

Figure. S3 Astrocyte-specific targeting NFAT5 alleviates astrocyte swelling and neuropathic pain

(A1-A2) Representative fluorescence images of Calcein AM signals and relative mean fluorescence intensity statistics in CTX-TNA2 cells with indicated treatments. LPS + Vehicle vs Control + Vehicle, $^{**}p < 0.01$, LPS + Vehicle vs LPS + KRN2, $^{*}p < 0.05$, $n = 3$, one-way ANOVA followed by Tukey's multiple comparison test. **(B1)** Schematic diagram of intra-spinal injection of astrocytes-specific AAV2/5 knockdown of NFAT5 (AAV2/5-sh*Nfat5*). **(B2)** Transfected fluorescent images after intra-spinal injection of

AAV2/5-sh*Nfat5*. Scale bar = 100 μm. **(B3)** Knockdown efficiency of NFAT5 after intra-spinal injection of AAV2/5-sh*Nfat5*. $*p < 0.05$, $n = 5$, two-tailed unpaired student's t-test. **(C1-C2)** Behavioral tests of PWMT and PWTL after intra-spinal injection of AAV2/5-sh*Nfat5*. $*p < 0.05$, $n = 8$, two-way ANOVA with repeated measures followed by Tukey's multiple comparison test. **(D1)** Representative 3D orthogonal confocal images of GFAP-stained astrocytes in each treatment group of rats SDH after intra-spinal injection of AAV2/5-sh*Nfat5*. Scale bar = 100 μm. **(D2-D3)** Morphological changes (average processes length and cell volume) of GFAP-stained astrocytes in indicated treatment group of rats SDH. $**p < 0.01$, $***p < 0.001$, $n = 30$ (3 rats per group, and 10 typical cells per rat were analyzed), two-tailed unpaired student's t-test.

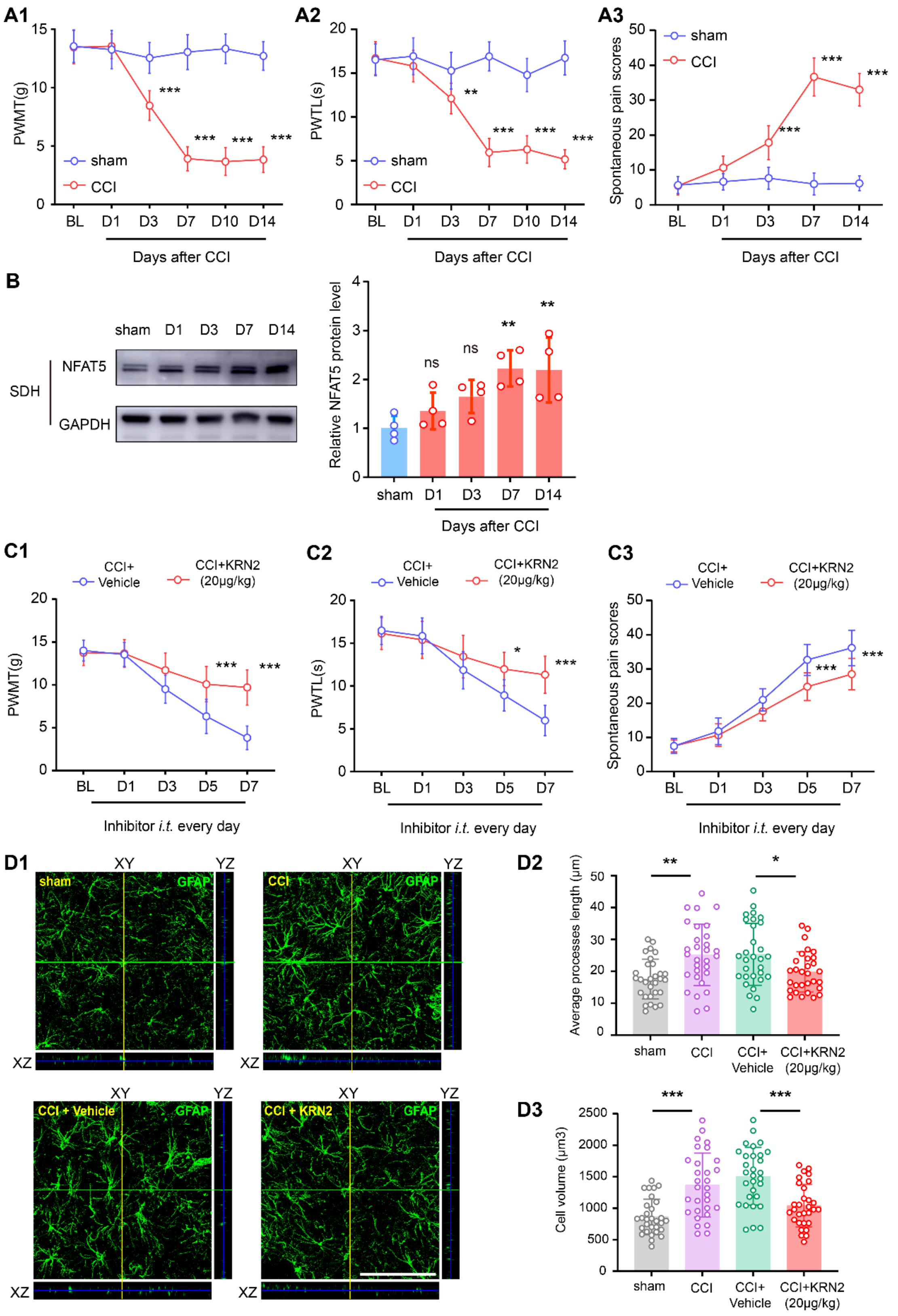

Figure. S4 Targeting NFAT5 alleviates astrocyte swelling and neuropathic pain in female rats.

(A1-A3) Behavioral tests of PWMT, PWTL and spontaneous pain scores in female rats

after CCI surgery. $^{**}p < 0.01$, $^{***}p < 0.001$, $n = 8$, two-way ANOVA with repeated measures followed by Tukey's multiple comparison test. **(B)** NFAT5 protein level in the injured ipsilateral SDH after sham or CCI surgery in female rats. $^{**}p < 0.01$, n = 4, one-way ANOVA followed by Tukey's multiple comparison test. **(C1-C3)** Behavioral tests of PWMT, PWTL and spontaneous pain scores in female rats after daily *i.t.* administration of NFAT5 inhibitor KRN2 over 7 days after CCI modeling. CCI + KRN2 (20 μg/kg) vs CCI + Vehicle, $^{*}p < 0.05$, $^{***}p < 0.001$, $n = 8$, two-way ANOVA with repeated measures followed by Tukey's multiple comparison test. **(D1)** Representative 3D orthogonal confocal images of GFAP-stained astrocytes in indicated group of female rats' SDH. Scale bar = 100 μm. **(D2-D3)** Morphological changes (average processes length and cell volume) of astrocytes. CCI vs sham, $^{**}p < 0.01$, $^{***}p < 0.001$, CCI + KRN2 (20 μg/kg) vs CCI + Vehicle, $^{*}p < 0.05$, $^{***}p < 0.001$, $n = 30$ (3 rats per group, and 10 typical cells per rat were analyzed), one-way ANOVA followed by Tukey's multiple comparison test.

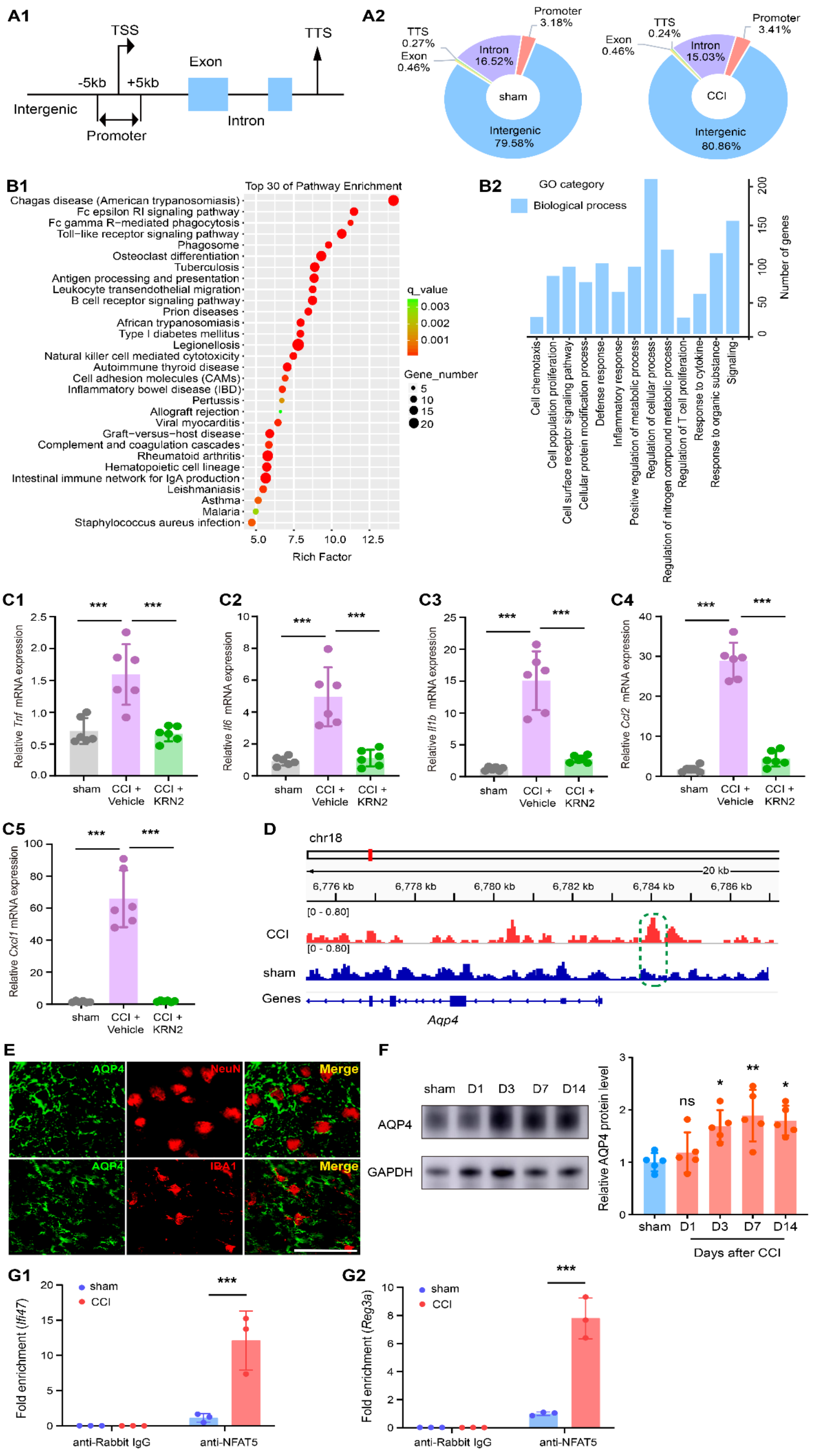
A1
TSS
TTS
Exon
-5kb
+5kb
Intergenic
Intron
Promoter
A2
Promoter 3.18%
TTS 0.27%
Exon 0.46%
Intron 16.52%
sham
Intergenic 79.58%
TTS 0.24%
Promoter 3.41%
Exon 0.46%
Intron 15.03%
CCI
Intergenic 80.86%
B1
Top 30 of Pathway Enrichment
Chagas disease (American trypanosomiasis)
Fc epsilon RI signaling pathway
Fc gamma R−mediated phagocytosis
Toll−like receptor signaling pathway
Phagosome
Osteoclast differentiation
Tuberculosis
Antigen processing and presentation
Leukocyte transendothelial migration
B cell receptor signaling pathway
Prion diseases
African trypanosomiasis
Type I diabetes mellitus
Legionellosis
Natural killer cell mediated cytotoxicity
Autoimmune thyroid disease
Cell adhesion molecules (CAMs)
Inflammatory bowel disease (IBD)
Pertussis
Allograft rejection
Viral myocarditis
Graft−versus−host disease
Complement and coagulation cascades
Rheumatoid arthritis
Hematopoietic cell lineage
Intestinal immune network for IgA production
Leishmaniasis
Asthma
Malaria
Staphylococcus aureus infection
Rich Factor
q_value
Gene_number
B2
GO category
Biological process
Number of genes
Cell chemotaxis
Cell population proliferation
Cell surface receptor signaling pathway
Cellular protein modification process
Defense response
Inflammatory response
Positive regulation of metabolic process
Regulation of cellular process
Regulation of nitrogen compound metabolic process
Regulation of T cell proliferation
Response to cytokine
Response to organic substance
Signaling
C1
Relative Tnf mRNA expression
C2
Relative Il6 mRNA expression
C3
Relative Il1b mRNA expression
C4
Relative Ccl2 mRNA expression
C5
Relative Cxcl1 mRNA expression
sham
CCI + Vehicle
CCI + KRN2
D
chr18
20 kb
6,776 kb
6,778 kb
6,780 kb
6,782 kb
6,784 kb
6,786 kb
[0 - 0.80]
CCI
sham
Genes
Aqp4
E
AQP4
NeuN
Merge
IBA1
F
sham D1 D3 D7 D14
AQP4
GAPDH
Relative AQP4 protein level
Days after CCI
ns
G1
sham
CCI
Fold enrichment (Ifi47)
anti-Rabbit IgG
anti-NFAT5
G2
Fold enrichment (Reg3a)

Figure. S5 NFAT5 dependent regulation in astrocytes.

(A1) Schematic diagram illustrating gene structures used in ChIP-seq of NFAT5 analysis. **(A2)** distribution of ChIP-seq peaks of NFAT5 binding sites in sham group and CCI group. Promoter = TSS ± 5 kb. **(B1)** KEGG enrichment analysis of differentiated expressed genes in the RNA-seq of sham and CCI rats. **(B2)** Gene Ontology (GO) enrichment analysis of differentiated expressed genes in the RNA-seq of sham and CCI rats. **(C1-C5)** Indicated mRNA expression in the SDH of CCI rats after daily intrathecal administration of NFAT5 inhibitor. CCI vs sham, $***p < 0.001$, CCI + KRN2 vs CCI + Vehicle, $***p < 0.001$, $n = 6$, one-way ANOVA followed by Tukey's multiple comparison test. **(D)** Genome browser view of NFAT5 enrichment peaks at the *Aqp4* locus in sham and CCI groups (sham: bule. CCI: red). **(E)** Immunofluorescence double-labeling of AQP4 (green) and indicated cell markers (NeuN or IBA1: red) in the SDH on day 7 after CCI modeling. Scale bar = 100 μm. **(F)** Expression of AQP4 protein in the injured ipsilateral SDH after sham or CCI surgery. $*p < 0.05$, $**p < 0.01$, $n = 5$, one-way ANOVA followed by Tukey's multiple comparison test. **(G1-G2)** ChIP-qPCR analysis of endogenous NFAT5 binding at *Ifi47* and *Reg3a* promoters in the SDH after CCI. $***p < 0.001$, $n = 3$, two-tailed unpaired student's t-test.

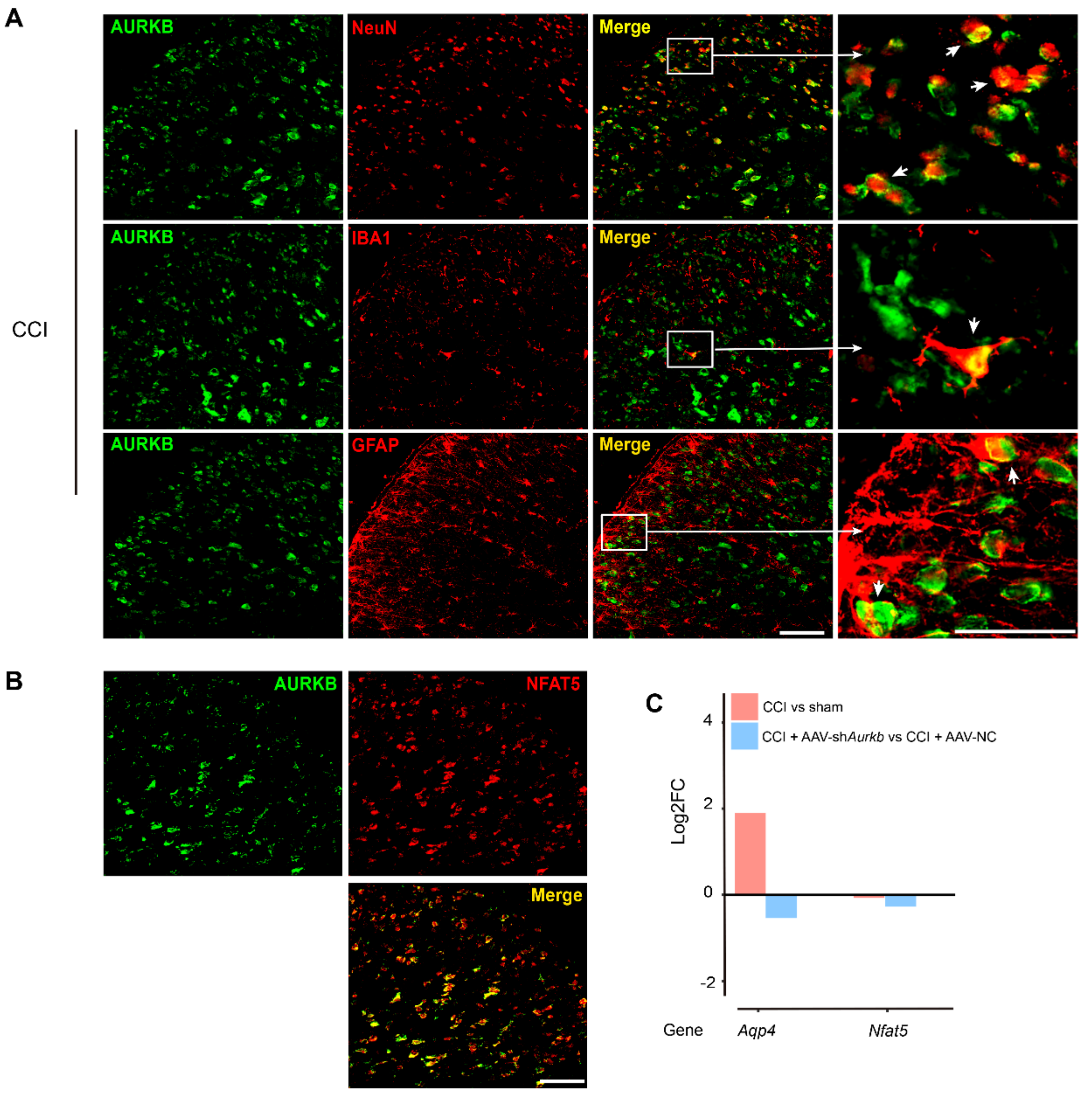

Figure. S6 Distribution of AURKB in the SDH and its downstream gene expression.

(A) Immunofluorescence double-labeling of AURKB (green) and indicated cell markers (NeuN, IBA1, GFAP: red) in the SDH on day 7 after CCI surgery. Scale bar = 100 μm. **(B)** Double immunofluorescence staining of AURKB (green) and NFAT5 (red) in the SDH on day 7 after CCI modeling. Scale bar = 100 μm. **(C)** The changed expression of *Aqp4* and *Nfat5* in the indicated rats. Log2FC: log2 fold change.

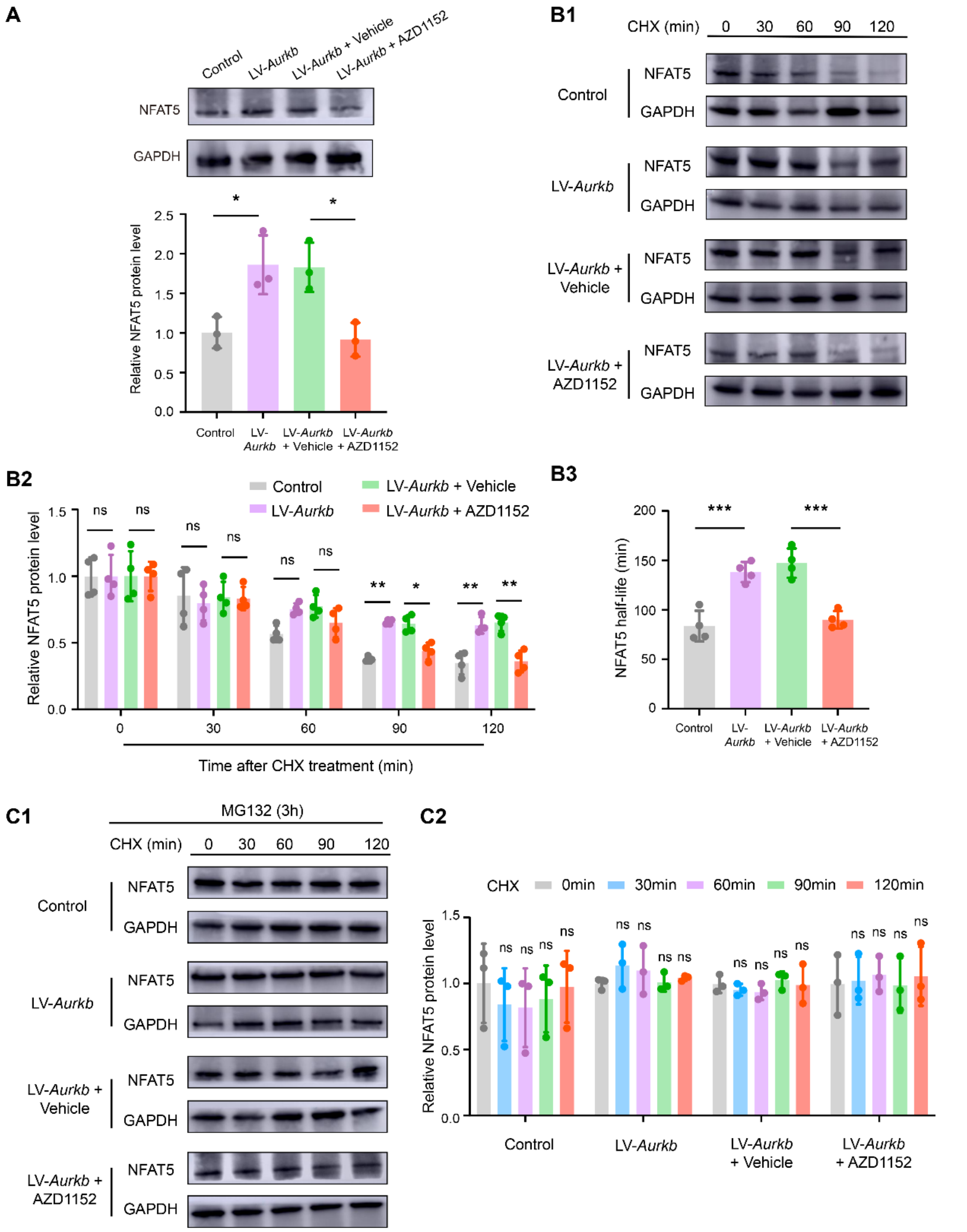

Figure. S7 AURKB maintains NFAT5 protein stability by restricting its protein degradation.

(A) Western blot analysis of NFAT5 in AURKB over-expressed HEK293T cells. Following transfection with LV-*Aurkb* for 72 hours, HEK293T cells were treated with the AURKB inhibitor AZD1152 for an additional 24 hours. Subsequently, Western blotting was employed to assess the total protein expression of NFAT5. Control vs LV-

Aurkb, *$p < 0.05$, LV-*Aurkb* + Vehicle vs LV-*Aurkb* + AZD1152, *$p < 0.05$, $n = 3$, one-way ANOVA followed by Tukey's multiple comparison test. **(B1)** Time-course analysis of NFAT5 protein levels. **(B2)** Normalized NFAT5 protein levels with GAPDH at indicated time points after CHX treatment. Control vs LV-*Aurkb*, **$p < 0.01$, LV-*Aurkb* + AZD1152 vs LV-*Aurkb* + Vehicle, *$p < 0.05$, **$p < 0.01$, $n = 4$, two-way ANOVA followed by Tukey's multiple comparison test. **(B3)** NFAT5 half-life in the indicated treated cells. Control vs LV-*Aurkb*, ***$p < 0.001$, LV-*Aurkb* + AZD1152 vs LV-*Aurkb* + Vehicle, ***$p < 0.001$, $n = 4$, one-way ANOVA followed by Tukey's multiple comparison test. **(C1-C2)** Time course analysis of NFAT5 protein levels in cells treated with proteasome inhibitor MG132.

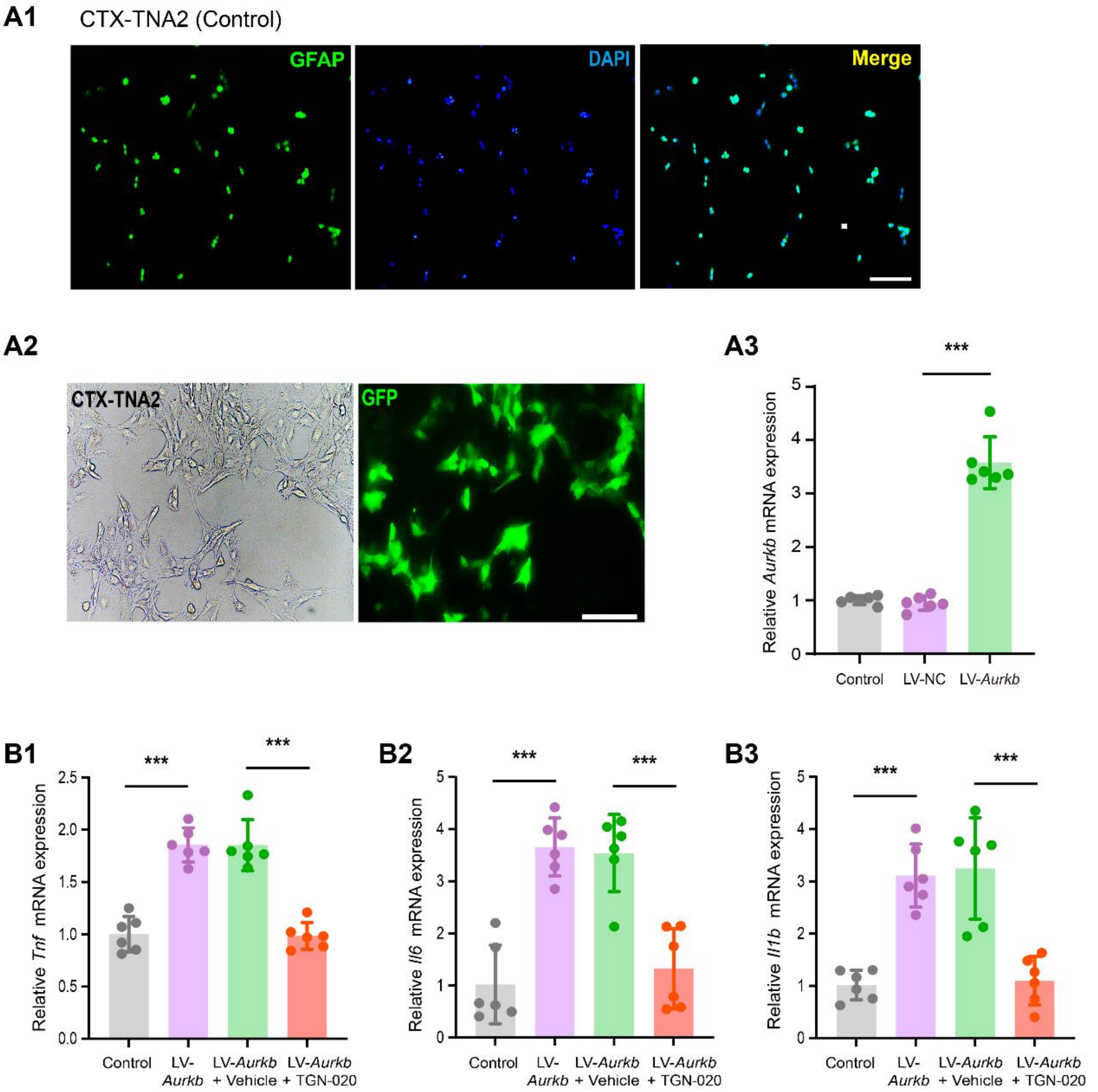

Figure. S8 AURKB mediates astrocyte associated inflammation *in vitro*.

(A1) GFAP immunofluorescence staining in CTX-TNA2 cell line. Scale bar = 100 μm. **(A2)** Immunofluorescence staining of overexpressed lentivirus LV-*Aurkb* (GFP) in CTX-TNA2 cells. Scale bar=100 μm. **(A3)** *Aurkb* mRNA level in LV-*Aurkb* transfected CTX-TNA2 cells. LV-*Aurkb* vs LV-NC, ***p < 0.001, n = 6, one-way ANOVA followed by Tukey's multiple comparison test. **(B1-B3)** The mRNA level of indicated inflammatory factors in LV-*Aurkb* transfected CTX-TNA2 cells administered with AQP4 inhibitor TGN-020. Control vs LV-*Aurkb*, ***p < 0.001, LV-*Aurkb* + Vehicle vs LV-*Aurkb* + TGN-020, ***p < 0.001, n = 6, one-way ANOVA followed by Tukey's multiple comparison test.

Table S1. The primer sequences involved in the experiment.

Gene	Primer Sequence
Nfat5	Forward 5' - GGCTCCCTTCCTGCTAATCCAATG - 3'<br>Reverse 5' - GCTGCTGCTGCTGTTGTTGTTG - 3'
Aqp4	Forward 5' - AGTCCAAAGCAGAGGGAGATGAGG - 3'<br>Reverse 5' - GAAGGCGGTCACAGCAGAGTTC - 3'
Actb	Forward 5' - CATCCTGCGTCTGGAACCTGG - 3'<br>Reverse 5' - TAATGTCACGCACGATTTCC - 3'
Tnf	Forward 5' - AAAGGACACCATGAGCACGGAAAG - 3'<br>Reverse 5' - CGCCACGAGCAGGAATGAGAAG - 3'
Il1b	Forward 5' - AATCTCACAGCAGCATCTCGACAAG - 3'<br>Reverse 5' - TCCACGGGCAAGACATAGGTAGC - 3'
Il6	Forward 5' - AGTTGCCTTCTTGGGACTGATGTTG - 3'<br>Reverse 5' - GGTATCCTCTGTGAAGTCTCCTCTCC - 3'
Ccl2	Forward 5' - CTCACCTGCTGCTACTCATTCACTG - 3'<br>Reverse 5' - CCTGCTGCTGGTGATTCTCTTGTAG - 3'
Cxcl1	Forward 5' - CCAATGAGCTGCGCTGTCAGTG - 3'<br>Reverse 5' - TGGCGGCATCACCTTCAAACTC - 3'

Table S2. NFAT5-related information in the single cell database.

Disease	Tissue	Cell Type	Gene	Variable Name	Value	Paper ID(PMID)
Alzheimer Disease (AD)	Prefrontal Cortex (PFC)	Excitatory neurons	NFAT5	logFC	0.04	31042697
Multiple Sclerosis (MS)	Cortical and subcortical lesion	Astrocytes	NFAT5	logFC	1.11	31316211
Age-related Macular Degeneration (AMD)	Eye	Microglia	NFAT5	logFC	-0.03	31653841

Table S3. ChIP-seq quality control related data.

Samples ID	Raw reads	Raw bases	Clean reads	Clean bases	Average length	Clean reads %	Clean bases%
CCI 1	28526414	4278962100	28194908	3889287028	137.94	98.84%	90.89%
CCI 2	60121576	9018236400	59292510	8174587188	137.87	98.62%	90.65%
CCI 3	62107278	9316091700	61392942	8465407506	137.89	98.85%	90.87%
sham 1	53266578	7989986700	51961156	7026484153	135.23	97.55%	87.94%
sham 2	30210688	4531603200	29469620	3973252910	134.83	97.55%	87.68%
sham 3	67155992	10073398800	65294528	8815474162	135.01	97.23%	87.51%

Table S4. RNA-seq quality control related data.

Samples ID	All reads	Mapped reads	Mapped pair reads	Mapped broken pair reads	Mapped unique reads	Mapped multi reads	Mapping ratio
CCI 1	37717090	35637363	34936900	700463	35480859	156504	94.49%
CCI 2	36260590	34272910	33554580	718330	34111183	161727	94.52%
CCI 3	46417410	43818381	42980918	837463	43633387	184994	94.40%
sham 1	39029134	36887910	36175954	711956	36732722	155188	94.51%
sham 2	44900980	42450711	41637324	813387	42272597	178114	94.54%
sham 3	41063422	38755091	38006298	748793	38590472	164619	94.38%

Table S5. The detailed information of 17 related genes overlapped by the results of ChIP-seq and RNA-seq.

Chr	Start	End	Peak score	Promoter (+/-5K)	Distance to TSS	Nearest promoter ID	Gene name
chr18	6,783,875	6,784,783	4.7	Yes	1118	NM_012825	*Aqp4*
chr17	46077832	46078112	4.52	No	37054	NM_001191940	*Aoah*
chr2	93859909	93860189	4.43	No	32545	NM_001109514	*Pmp2*
chr4	109473078	109473358	3.91	Yes	-4675	NM_001145846	*Reg3a*
chr4	3153437	3153717	3.74	No	110346	NM_012589	*Il6*
chr9	52093252	52093532	3.65	No	70097	NM_032085	*Col3a1*
chr1	198817943	198818223	3.56	No	73891	NM_001033998	*Itgal*
chr4	155984724	155985004	3.56	No	-24955	NM_001005890	*Clec4a1*
chr17	28158082	28158362	3.56	No	20660	NM_001106128	*Ly86*
chr10	34278510	34278790	3.56	Yes	657	NM_172019	*Ifi47*
chr9	52107109	52107389	3.48	No	83954	NM_032085	*Col3a1*
chr13	60993624	60993904	3.48	No	76758	NM_019336	*Rgs1*
chr17	46307761	46308041	3.48	No	-192875	NM_001191940	*Aoah*
chr17	46276974	46277254	3.48	No	-162088	NM_001191940	*Aoah*
chr17	46031308	46031588	3.48	No	83578	NM_001191940	*Aoah*
chr2	104593827	104594107	3.39	No	-132104	NM_031019	*Crh*
chr2	104509443	104509723	3.39	No	-47720	NM_031019	*Crh*
chr2	192657445	192657725	3.39	No	13474	NM_021864	*Sprrla*
chr4	109313986	109314266	3.39	No	-153146	NM_053289	*Reg3b*
chr2	104509723	186592916	3.3	No	12316	NM_001107702	*Fcrl2*
chr4	43696143	43696423	3.3	No	440532	NM_022379	*Tfec*
chr7	54787681	54787961	3.3	No	-8998	NM_001011987	*Glipr1*
chr4	43638966	43639246	3.22	No	497709	NM_022379	*Tfec*
chr9	52053131	52053411	3.22	No	29976	NM_032085	*Cal3a1*

Table S6. Ingenuity Pathway Analysis (IPA) results based on transcriptome sequencing data.

IPA group	Upstream regulator	Molecule type	Activation z-score
CCI + AAV-sh*Aurkb* vs CCI + AAV-NC	NFAT5	transcription regulator	-3.988
